# Synthesis of octagon-containing molecular nanocarbons

**DOI:** 10.1039/d1sc05586k

**Published:** 2021-12-13

**Authors:** Greco González Miera, Satoshi Matsubara, Hideya Kono, Kei Murakami, Kenichiro Itami

**Affiliations:** Institute of Transformative Bio-Molecules (WPI-ITbM) and Graduate School of Science, Nagoya University Chikusa Nagoya 464-8602 Japan itami@chem.nagoya-u.ac.jp; Department of Chemistry, School of Science, Kwansei Gakuin University Sanda Hyogo 669-1337 Japan kei.murakami@kwansei.ac.jp; JST-PRESTO 7 Gobancho, Chiyoda Tokyo 102-0076 Japan; Institute of Chemistry, Academia Sinica Nankang Taipei 115 Taiwan Republic of China

## Abstract

Nanocarbons, such as fullerenes, carbon nanotubes, and graphenes, have long inspired the scientific community. In order to synthesize nanocarbon molecules in an atomically precise fashion, many synthetic reactions have been developed. The ultimate challenge for synthetic chemists in nanocarbon science is the creation of periodic three-dimensional (3D) carbon crystals. In 1991, Mackay and Terrones proposed periodic 3D carbon crystals with negative Gaussian curvatures that consist of six- and eight-membered rings (the so-called Mackay–Terrones crystals). The existence of the eight-membered rings causes a warped nanocarbon structure. The Mackay–Terrones crystals are considered a “dream material”, and have been predicted to exhibit extraordinary mechanical, magnetic, and optoelectronic properties (harder than diamond, for example). To turn the dream of having this wonder material into reality, the development of methods enabling the creation of octagon-embedding polycyclic structures (or nanographenes) is of fundamental and practical importance. This review describes the most vibrant synthetic achievements that the scientific community has performed to obtain curved polycyclic nanocarbons with eight-membered rings, building blocks that could potentially give access as templates to larger nanographenes, and eventually to Mackay–Terrones crystals, by structural expansion strategies.

## Abstract 2

Nanocarbons, such as fullerenes, carbon nanotubes, and graphenes, have long inspired the scientific community.

## Graphical abstract

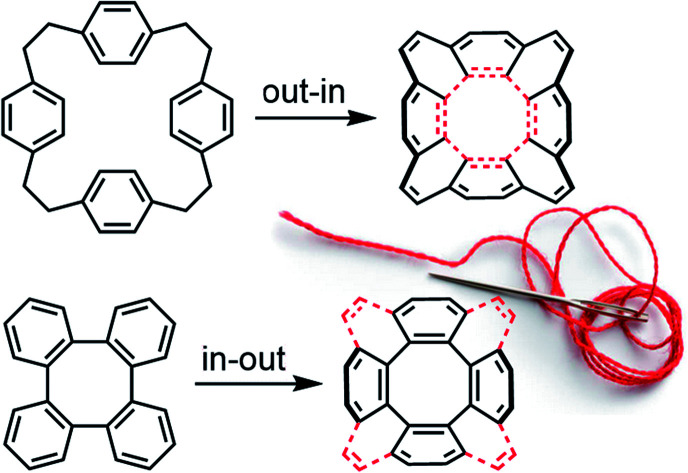

## 1 Introduction

**Fig. 1:**
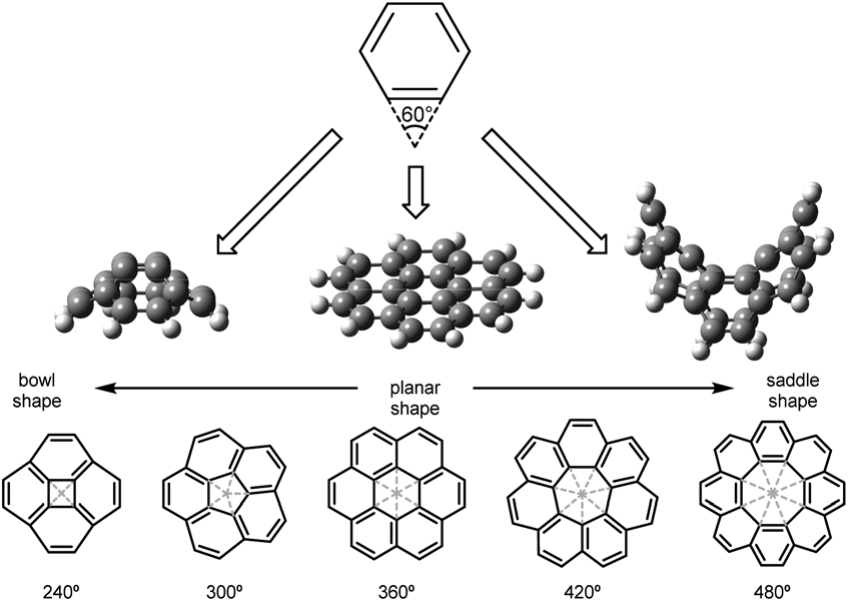
Wedge angles and nanographenes with positive, zero and negative Gaussian curvature.^4^

The family of sp^2^ carbon allotropes displays different topologies and their properties depend on the π-conjugated electronic structure.^1^ These modifications or defects can be caused by the inclusion of heteroatoms within the nanocarbon lattice.^2^ Alternatively, polycyclic aromatic hydrocarbons (PAHs) can be cataloged, depending on the absence or presence of geometric defects in the hexagonal graphene layer, in planar and nonplanar, respectively. Planar six-membered lattices show zero Gaussian curvature. Curvature can be positive with the inclusion of four- or five-membered rings. When seven- or eight-membered rings are present within the carbon scaffold, the curvature is negative. The polygonal curvature is predicted by cutting each ring into triangles and summing up their interior angles, or wedge angles. Values smaller than 360° correspond to PAHs or nanographenes with positive curvature that display bowl shape, observed in four- and five-membered rings. On the other hand, typical examples of rings with negative curvature and saddle/tub shape are seven-, eight-membered rings, and beyond, with angle values larger than 360° (Fig. 1).^3^

Thus, we can observe that Gaussian curvatures arise from the geometry of the surface in an sp^2^ carbon lattice, and not from the intrinsic shape of the sp^2^-carbon allotrope. Graphene, carbon nanotubes, carbon nanorings, and carbon nanobelts have all in common a zero Gaussian curvature, although only graphene is a planar carbon sheet. This family of PAHs is solely composed of fused benzenoid rings, hence the sum of the internal triangles of every six-membered ring is nearly 360° and bending the carbon sheet along a line does not disrupt the zero-curvature.

**Fig. 2:**
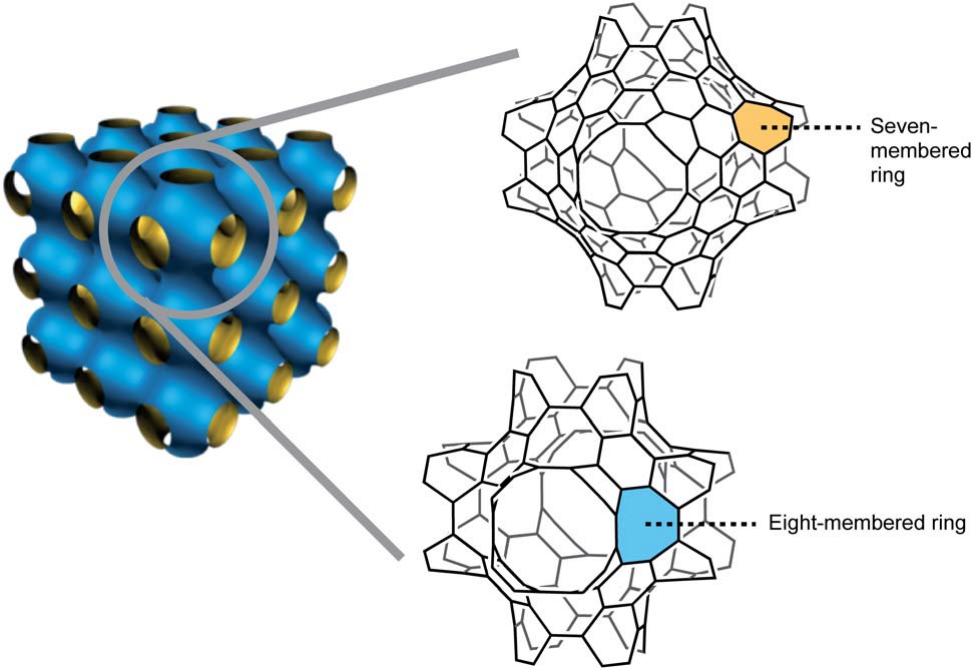
Negatively curved polycyclic aromatic hydrocarbons (PAHs) as building blocks of carbon schwarzites.

The macrocyclic expansion of polycyclic systems with non-zero curvature gives additional three-dimensional sp^2^ scaffolds, such as spherical and positively curved fullerene that features five-membered rings, and the synthetically elusive carbon schwarzite,^5–7^ proposed by Mackay and Terrones,^8^ a polycyclic network structure with negative Gaussian curvature that can contain seven- and eight-membered rings (Fig. 2). Additionally, other hypothetical allotropes feature positive and negative curvatures, such as toroidal carbon nanotubes. This review describes the most vibrant synthetic achievements that the scientific community has performed to obtain curved PAHs with eight-membered rings, building blocks that could potentially give access as templates to larger nanographenes, and eventually to Mackay–Terrones crystals, by structural expansion strategies.

## 2 Synthesis of building blocks

Carbon schwarzites were named after H. A. Schwarz, the German mathematician who proposed periodic minimal surfaces that can describe these hypothetical curved hydrocarbons.^9^ With a topology that features heptagons and octagons within a graphitic lattice, “defects” that distance them from planar graphene, they were first proposed by Mackay and Terrones.^8^ Carbon schwarzites, also known as Mackay–Terrones crystals have been hypothesized but they are yet to be synthesized.

Bottom-up synthetic methods represent an ambitious challenge but they have proven successful in giving access to nanographenes with defined structures.^10^ For the synthesis of carbon schwarzites, [*n*]circulene structures are clear candidates as building blocks. These PAHs display a central ring of *n*-sides enclosed with the same number of *ortho*-annulated aromatic rings. When the central cavity is surrounded only with benzenoid rings, these macrocyclic aromatic systems show characteristic shapes upon changing the size of the central hub; bowl shape for [4]circulene^11^ and [5]circulene,^12–14^ planar for [6]circulene,^15^ and saddle for [7]circulene,^16,17^ [8]circulene and other hypothetical higher-order analogs. Once obtained the appropriate [*n*]circulenes, bottom-up approaches might give access to obtain larger curved polycyclic systems.

This review will focus on the synthesis of curved [8]circulene derivatives, macrocyclic arenes in which a central octagon is fully surrounded by eight-fused benzene rings, and its derivatives. As well as with cyclooctatetraene, the central eight-membered ring shows a tub shape that derives into a saddle 3D structure when peripheral rings are assembled around the core. The benzene rings that surround the central hub are distributed in an alternating in and out of the plane wave-shaped (curve-up and curve-down) pattern. Thus, the negative curvature present in saddle-shaped structures originates from this doubly-concave shape.

Planar [8]circulenes, such as [8]heteracirculenes and quasi-[8]heteracirculenes with five-membered rings around the central octagon and heteroatoms replacing etheno bridges,^2,18^ show planar structures and they therefore lie out of the focus of this review, centered in tub- and saddle-shaped structures with a [8]circulene core. To date, the synthesis of [8]circulene remains elusive. Early attempts by Wennerström and coworkers in 1976 did not yield the desired product, allegedly owing to a highly strained and unstable structure.^19^ Later calculations by Sansón and coworkers in 2004 confirmed the instability of [8]circulene rising from concentric aromatic currents between the inner and outer rings.^20^ Therefore, current synthetic strategies focus on related substituted structures that exhibit higher stability. The difference in stability between these two species can be explained through their aromaticity.

**Fig. 3:**
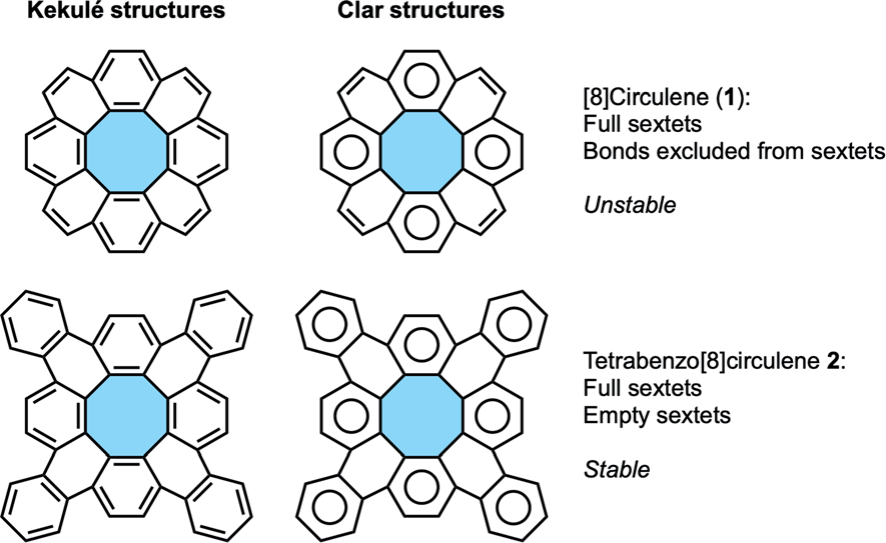
Aromaticity proposals according to Kekulé and Clar's theories for [8]circulene (1) and tetrabenzo[8]circulene 2.

According to Clar's aromatic sextet rule,^21,22^ [8]circulene (1) displays non-aromatic π-electrons, *i.e.* double bonds that are excluded from aromatic sextets.^23^ These double bonds are likely to be the source of instability, being reactive sites in PAH structures upon their inherent structural strain (Fig. 3). In tetrabenzo[8]circulene 2, the addition of peripheral benzenoid rings locates all electrons in aromatic sextets, rising the stability compared to the parent [8]circulene.

The current review will divide the synthesis of substituted [8]circulene scaffolds into two directional approaches. In the in–out cyclization strategy, the eight-membered core is prepared first and the outer benzenoid layer is subsequently intramolecularly “stitched” to afford the [8]circulene scaffold. Alternatively, the out–in cyclization strategy starts from a macrocycle that assembles the inner octagon in the last synthetic step. Additionally, to understand the in–out cyclization, the review will cover the main synthetic strategies of tetraphenylene derivative first, as precursors of [8]circulenes *via* later functionalization, and catalog them in terms of the nature of the starting material employed in each synthetic structure.

### 2.1. In–out cyclization strategy (intramolecular stitching)

**Fig. 4:**
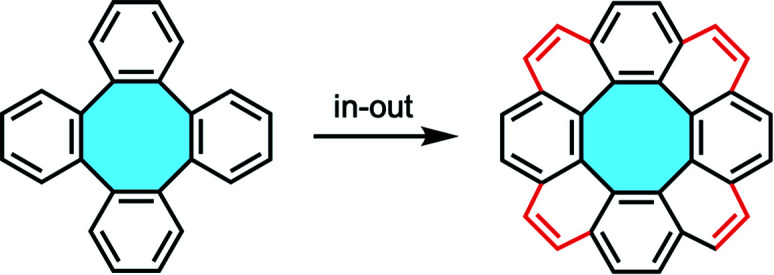
Schematic illustration of the in–out cyclization approach towards [8]circulene derivatives.

The most straightforward bottom-up approach towards eight-membered π-extended nanocarbons might involve the synthesis of tetraphenylenes, which display four benzene rings *ortho*-annulated to a central eight-membered ring. The formation of the cyclooctatetraene (COT) core has been vastly investigated by the scientific community, and it could be an ideal starting material to obtain [8]circulenes. These tube-shaped molecules might undergo intramolecular stitching of the outer ring, maintaining the original Gaussian curvature to afford fully-fused nanographenes with saddle shape and a central eight-membered ring (Fig. 4).

#### 2.1.1. Synthetic strategies to form the eight-membered ring central hub

As a molecule grows, the geometrical topology can be understood from its key units. Cyclooctatetraene is the most basic eight-membered ring and it features a tub shape that will extend along with the molecule, and any derivative will therefore adopt the extended saddle shape observed in polycyclic networks with negative Gaussian curvature. Tetraphenylene (3) displays a saddle-shaped structure with two opposite pairs of benzenoid rings alternating up and down the plane of a central cyclooctatetraene hub. Tetraphenylene can be considered as a valuable building block for [8]circulenes following intramolecular stitching methodologies, but this molecule and derivatives have also shown outstanding capabilities as liquid crystals,^24–26^ molecular devices,^27^ and chiral ligands in catalysis.^28–33^ In addition, the establishment of heteroatom bridges in tetraphenylene rings planarizes the scaffold, becoming potential candidates for electronic materials with new semiconductor properties.^34^

##### 2.1.1.1. Tetramerization of benzyne units

**Scheme 1:**
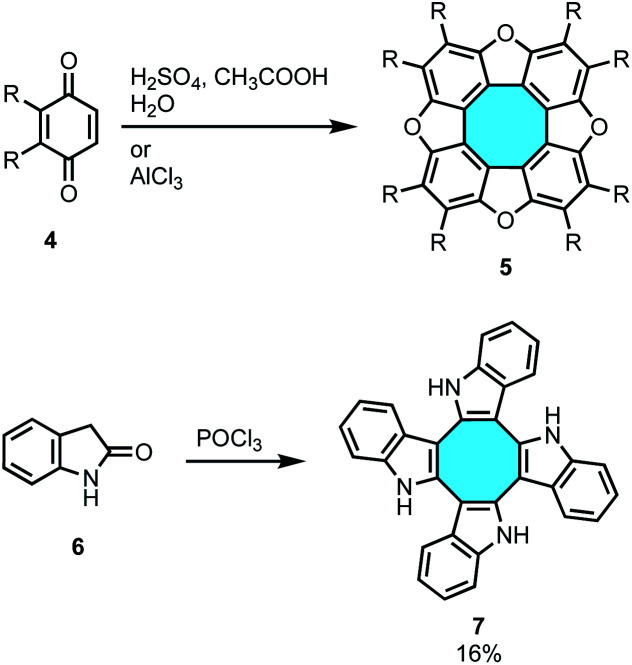
Tetramerization strategies towards eight-membered rings.

The simplest approach for the synthesis of tetraphenylene would entail the tetrameric condensation of four benzyne units, but efforts in this strategy have been unsuccessful to date. Nevertheless, tetramerization reactions of benzoquinones 4 and indolin-2-one 6 have yielded planar heteroatomic [8]circulenes such as tetraoxa[8]circulenes 5 and cyclooctatetraindoles 7, respectively (Scheme 1).^34–36^ Many recent publications have shown bright advances using alternative synthetic protocols that overcome this limitation in the construction of curved eight-membered ring frameworks.

##### 2.1.1.2. Coupling of biphenyls

**Scheme 2:**
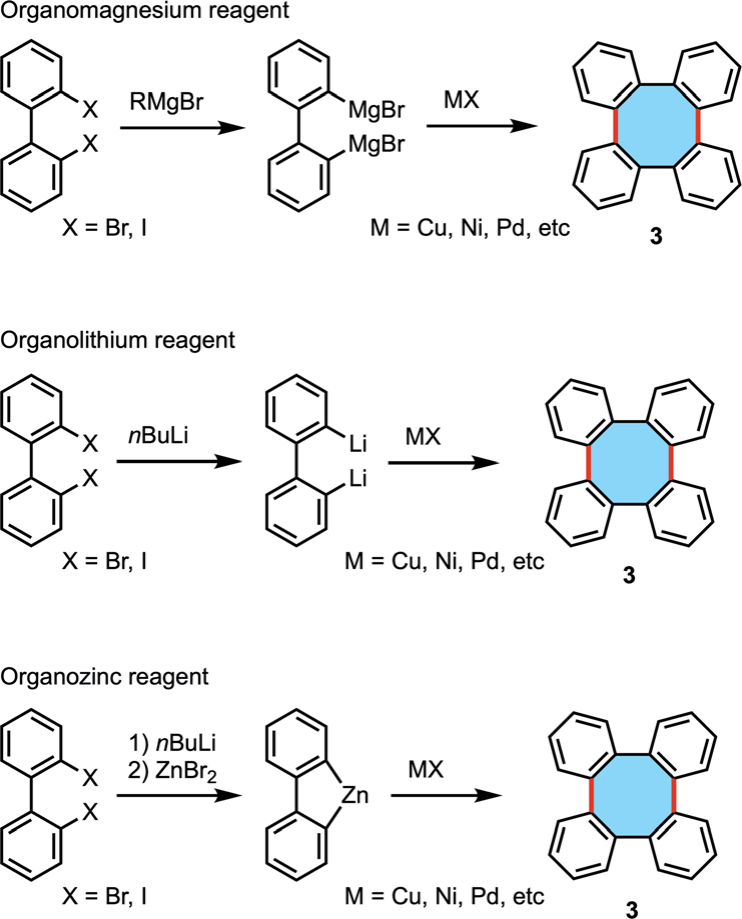
Oxidative coupling of organometallic compounds.

There is a plethora of dihalobiphenyls that can benefit from the widespread use of organometallic reagents, *e.g.* organomagnesium, -lithium, or -zinc compounds, in halogen–metal exchange and subsequently undergo homocoupling reactions (Scheme 2). The broad offer of synthetic protocols of tetraphenylenes *via* transmetallation has caught the attention of many researchers from as early as 1943. van Niekerk and coworkers obtained tetraphenylene for the first time, together with biphenylene, by means of the copper-catalyzed homocoupling reaction of an organomagnesium reagent previously prepared from 2,2′-dibromobiphenyl.^37^ Crystals of tetraphenylene (3) were found to be stable and they could be recovered by the action of potassium permanganate in boiling acetone. Early studies by Wittig and Klar showed progress on the synthesis of tetraphenylenes by homocoupling of dilithiobiphenyl with different transition metal chlorides.^38^ The group of Wong has performed large achievements in copper-catalyzed homocoupling reactions of dilithio substrates to afford substituted tetraphenylene scaffolds that can be further functionalized and even interconnected leading to more complex structures.^28,39–41^

In 2000, Rajca and coworkers obtained a chiral, nonracemic binaphthyl-derived tetraphenylene with 95% ee following the copper-mediated homocoupling of the corresponding dilithio monomer.^42^ To show the current relevance of these protocols, Kaku, Tsunoda, and coworkers recently obtained a substituted tetraphenylene *via* oxidative homocoupling after double lithiation of dibromo biphenyl that could be further enantiomerically enriched to be employed as chiral ligands.^43^ Kabir, Iyoda, and coworkers explored copper-mediated homocoupling reactions of organozinc reagents derived from dihalobiphenyls to yield tetraphenylenes. The formation of transient arylzinc intermediates was a key step prior transmetallation employing CuBr_2_.^44,45^

**Scheme 3:**
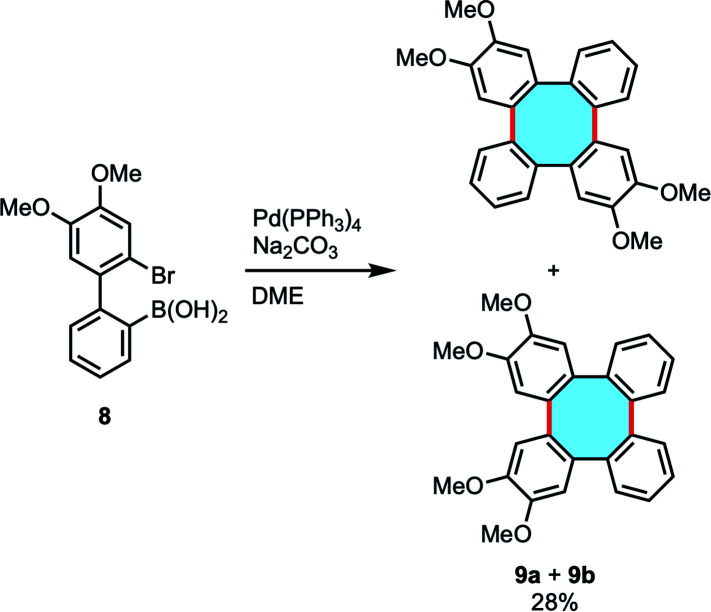
Pd-catalyzed intermolecular Suzuki–Miyaura coupling.

Lithium reagents offer a limited variety of substrates and the synthetic preparation of dilithio transient products can be labored, and many of these methods require stoichiometric amounts of lithium or copper reagents. Interestingly, biphenyl derivatives can also lead to tetraphenylenes by cyclodimerization reactions in catalytic conditions. The group of Wong investigated this strategy following a Pd-catalyzed intermolecular Suzuki–Miyaura coupling protocol of biaryl 8 with bromide and boronic acid functionalities (Scheme 3). The expected regio- and stereoselective synthesis of 2,3,10,11-tetramethoxytetraphenylene 9a did not take place, and instead an equimolar mixture of 9a and the isomer 2,3,6,7-tetramethoxytetraphenylene 9b was produced in low yield.^46^ Interestingly, the isolation of palladium complex intermediates was discussed as strong evidence for the existence of a palladacycle intermediate that accounts for the formation of isomers.

**Scheme 4:**
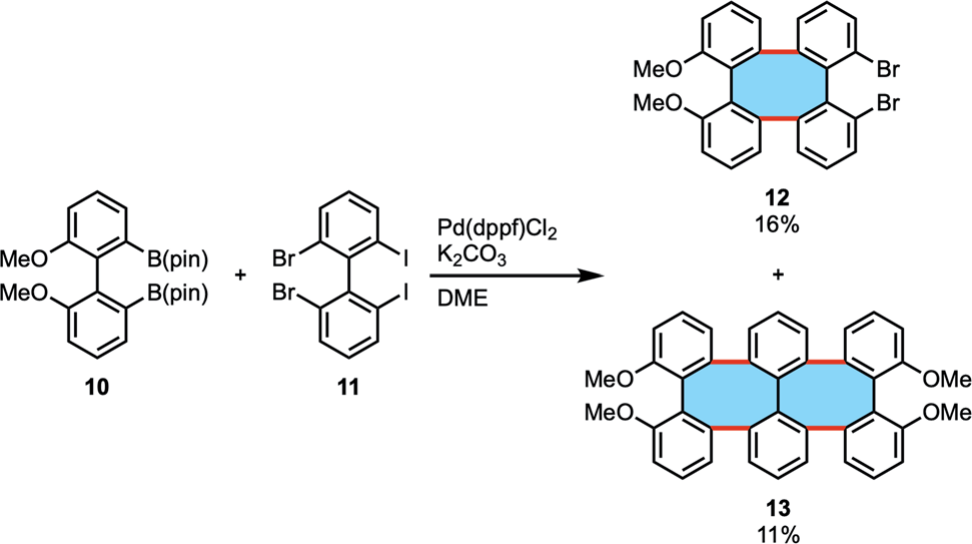
Double Suzuki–Miyaura coupling of biphenyl diborate 10 and dibromodiiodobiphenyl 11.

In an effort to synthesize helical oligomers containing eight-membered rings, Wong and coworkers attempted to use double Suzuki–Miyaura coupling of biphenyl diborate 10 and 2,2′-dibromo-6,6′-diiodo-1,1′-biphenyl (11) in the presence of Pd(dppf)Cl_2_.^47^ The results showed the formation of tetraphenylene 12 and compound 13 bearing six benzene rings and two eight-membered rings in 16% and 11% yields, respectively (Scheme 4). They subsequently attempted to apply the double Suzuki–Miyaura coupling to the synthesis of compounds with eight benzene rings, but were unable to obtain the desired compounds. They speculated that it was due to the sterically bulky structure. The target oligophenylene bearing eight benzene rings could be synthesized from the appropriate tetraphenylene *via* the halogen–lithium exchange and Cu(ii)-mediated homocoupling reaction sequence.

**Scheme 5:**
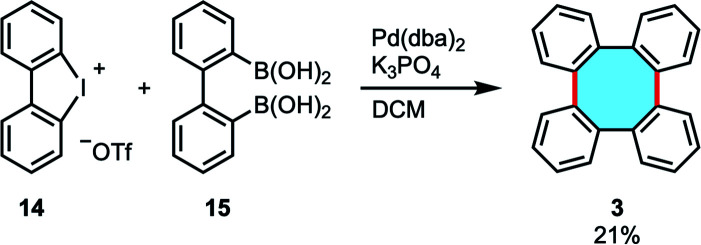
Pd-catalyzed double Suzuki–Miyaura reaction using cyclic dibenziodonium and biphenyldiboronic acid.

In a research work highlighting the Pd-catalyzed double Suzuki–Miyaura reaction of cyclic dibenziodonium salts 14 and biphenyldiboronic acids for the synthesis of *o*-tetraaryls, Wong and coworkers reported the synthesis of tetraphenylene (3) in low yield when they employed biphenyldiboronic acid 15 in the coupling reaction (Scheme 5).^48^

**Scheme 6:**
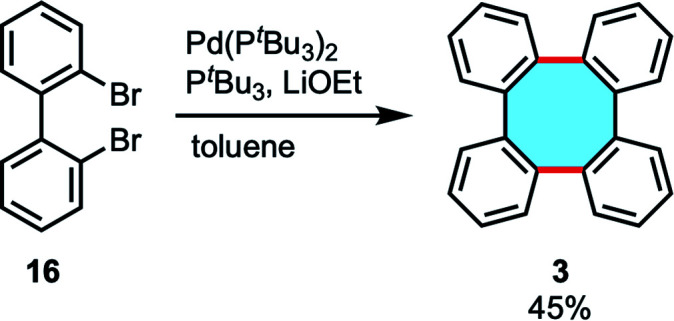
Ullmann-type cyclodimerization of biphenyl dibromides.

In 2015, Ouyang and Xi presented the first catalytic protocol for the cyclodimerization of dibromobiphenyl 16 catalyzed by palladium, obtaining tetraphenylene (3) in 45% yield (Scheme 6).^49^

**Scheme 7:**
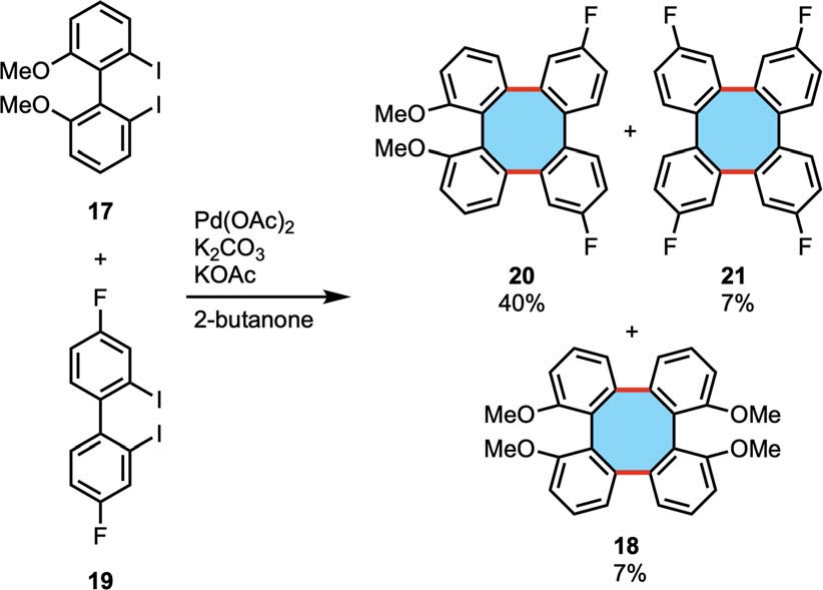
Pd-catalyzed double Ullmann-type coupling and cross-coupling of diiodobiaryls.

In 2016, Wong and coworkers reported the synthesis of tetraphenylenes by Pd-catalyzed double Ullmann-type coupling and cross-coupling reactions of diiodobiaryls (17 and 19).^50^ This protocol allowed accessing symmetric and unsymmetric substituted tetraphenylenes (18, 20, and 21) in low to moderate yields. In the cross-coupling examples, mixtures of three possible cross-coupling products and the homocoupling product were obtained, with the unsymmetrical adduct 20 being the major product (Scheme 7). The NMR and single-crystal X-ray diffraction analyses confirmed the 3D structures. Attempts to use enantiopure diiodobinaphthyl led to racemization of the corresponding tetraphenylene.

**Scheme 8:**
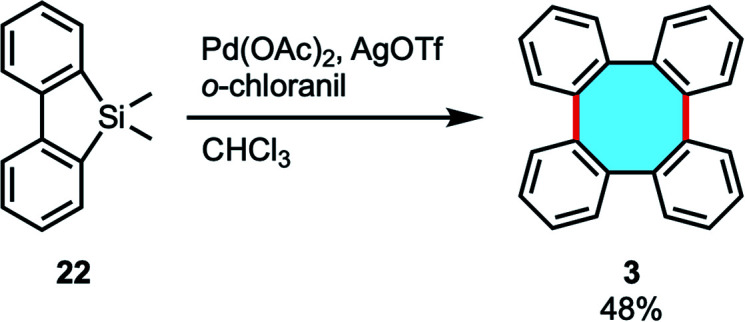
Pd-catalyzed oxidative homocoupling of arylsilanes.

In 2017, our group reported the palladium/*o*-chloranil-catalyzed oxidative homocoupling reaction using arylmethylsilanes towards biaryls (Scheme 8).^51^ This reaction was applied to 5,5-dimethyl dibenzosilole 22 to synthesize tetraphenylene (3) in 48% yield.

**Scheme 9:**
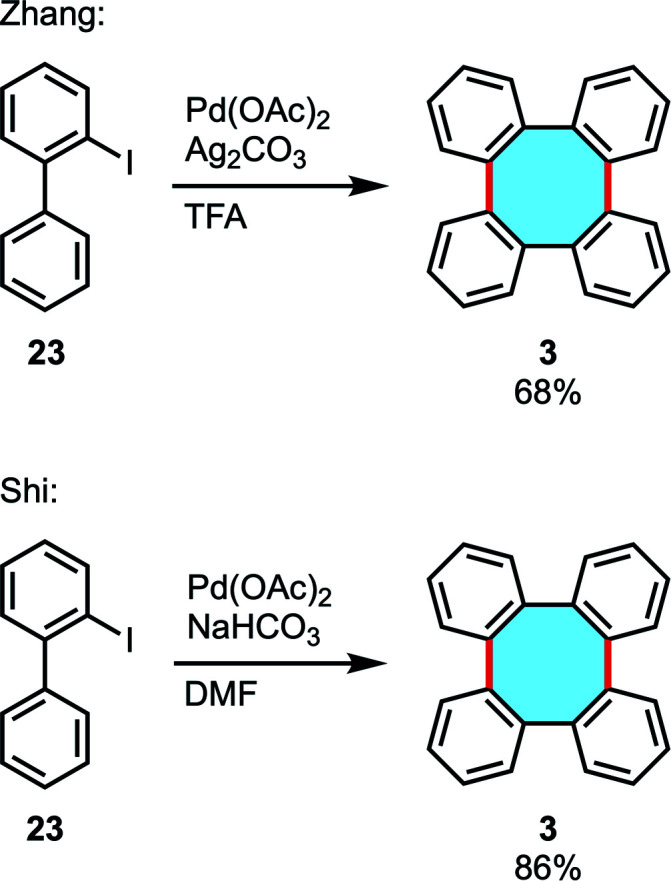
Pd-catalyzed C–H arylation of halobiaryls 23.

In 2016, the groups of Zhang and Shi independently reported the palladium-catalyzed cyclodimerization of iodobiphenyls 23 to give tetraphenylenes.^52,53^ A wide variety of substituted tetraphenylenes can be directly synthesized *via* dimerization of *o*-iodobiphenyls in medium to good yields (Scheme 9). These research works demonstrated the applicability of C–H functionalization to easily construct eight-membered rings and even in multigram scalable processes.

##### 2.1.1.3. Dimerization of biphenylene

**Scheme 10:**
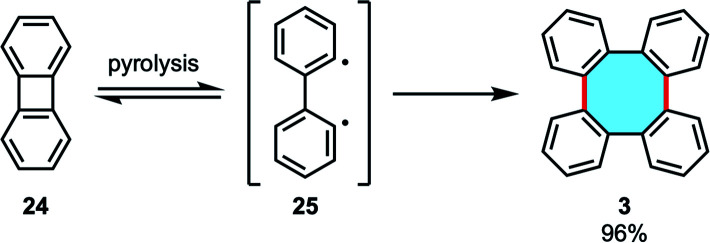
Pyrolysis of biphenylene leading the synthesis of 3.

Another option is to use the formal dimer of benzyne, biphenylene (24), to construct the skeleton of tetraphenylene. Many researchers have considered this building block in their methodologies, focusing either on ring-expansion homo-dimerizations or cross-dimerizations. Early dimerization studies by pyrolysis were reported by Friedman and coworkers.^54^ In the course of investigations to generate benzyne by electron impact from 24, they instead observed the formation of tetraphenylene (3) as the major product at 400 °C for 1 h in 96% yield (Scheme 10). Higher temperatures led to thermal decomposition, and lower temperatures afforded lower yields of 3 although conversions were high, allegedly from a competition between dimerization of diradical 25 and radical addition towards polymeric mixtures.

**Scheme 11:**
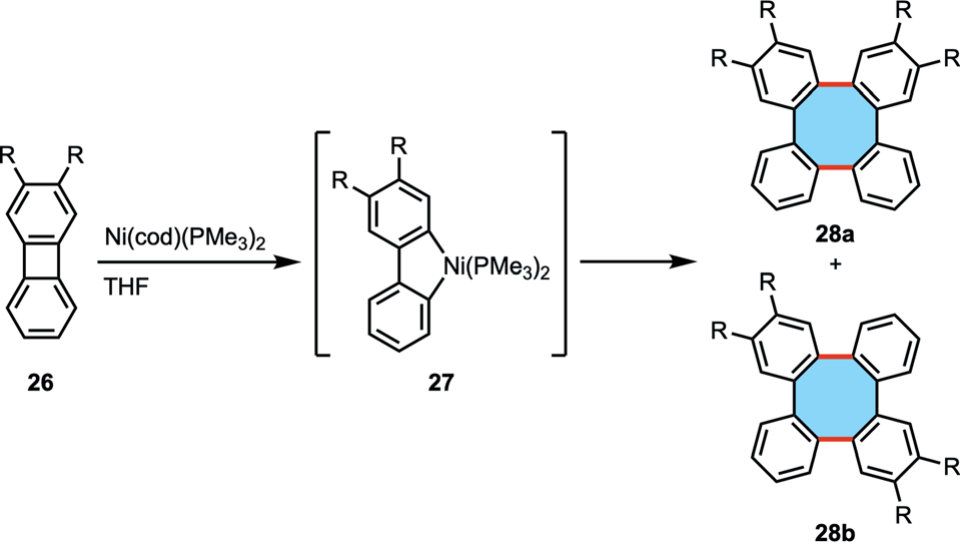
Nickel-catalyzed dimerization of biphenylenes.

Although they promote impressive yields in the formation of 3, pyrolysis processes required high temperatures and the scope was limited to unsubstituted products. The use of transition-metal catalysts in the dimerization of 24 was explored by the group of Watson in 1961, who reported the residual formation of tetraphenylene upon treatment with [Ni(CO)_2_(PPh_3_)_2_] in their studies for the synthesis of coordination complexes.^55^ Vollhardt and coworkers extended the study of the tandem transition-metal-catalyzed dimerization. The protocol allowed the use of substituted biphenylenes 26 albeit yielded equimolar mixtures of two possible isomers 28a and 28b in low yields (Scheme 11).^56^ Mechanistic investigations performed by Eisch and coworkers suggested that the dimerization mechanism might occur *via* the formation of a nickel–tetrabenzo complex that was characterized by X-ray crystal structure analyses.^57^ The groups of Jones and Radius have widely studied and expanded the scope of transition metal complexes that can readily catalyze the dimerization of biphenylene to nickel, platinum, palladium and rhodium in sequential C–C insertion reactions.^58–60^ The groups of Sharp, Johnson and Sanford deepened into the mechanistic insights of the transition-metal-catalyzed mechanism, but the limited availability of biphenylenes has probably hindered further developments of this strategy.^61–63^

**Scheme 12:**
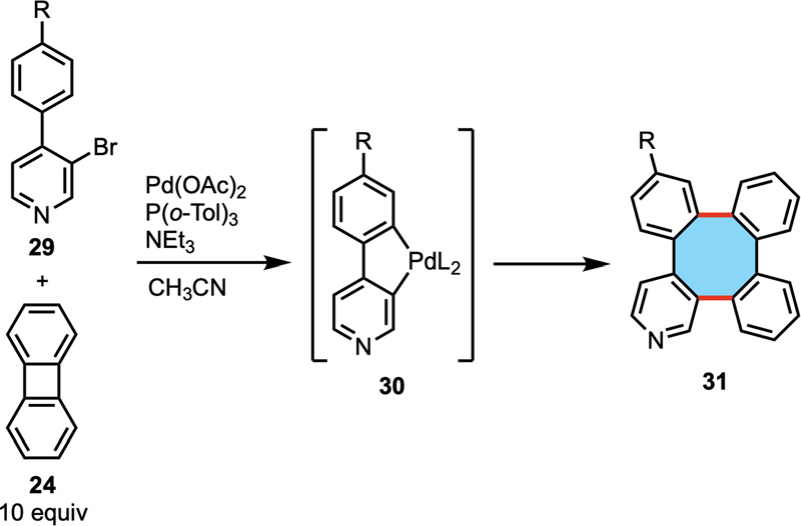
Pd-catalyzed cross-coupling of biaryl halides 29 and biphenylene.

In general terms, this strategy has several drawbacks, such as a limited variety of accessible products, which are either highly symmetric or mixtures of isomers. For that reason, the development of cross-coupling reactions was necessary for the efficient synthesis of a greater variety of tetraphenylene analogs. Many of the aforementioned works in biphenylene dimerization showed that the mechanism involves metallacycle intermediates, and Gallagher and coworkers published a study on Mizoroki–Heck reactions of biaryl halides with a palladacycle intermediate.^64^ This motivated them to study the possibility to use biaryls to access and to intercept the transient palladacycle intermediate needed in the homocoupling reaction of biphenylene, which would open new ways to synthesize unsymmetrical tetraphenylene derivatives (Scheme 12).^65^ In fact, the reaction of halobiaryls 29 with biphenylene 24 in the presence of Pd(OAc)_2_ afforded several heterocyclic tetraphenylenes 31*via* palladacycle 30, albeit in low yields.

**Scheme 13:**
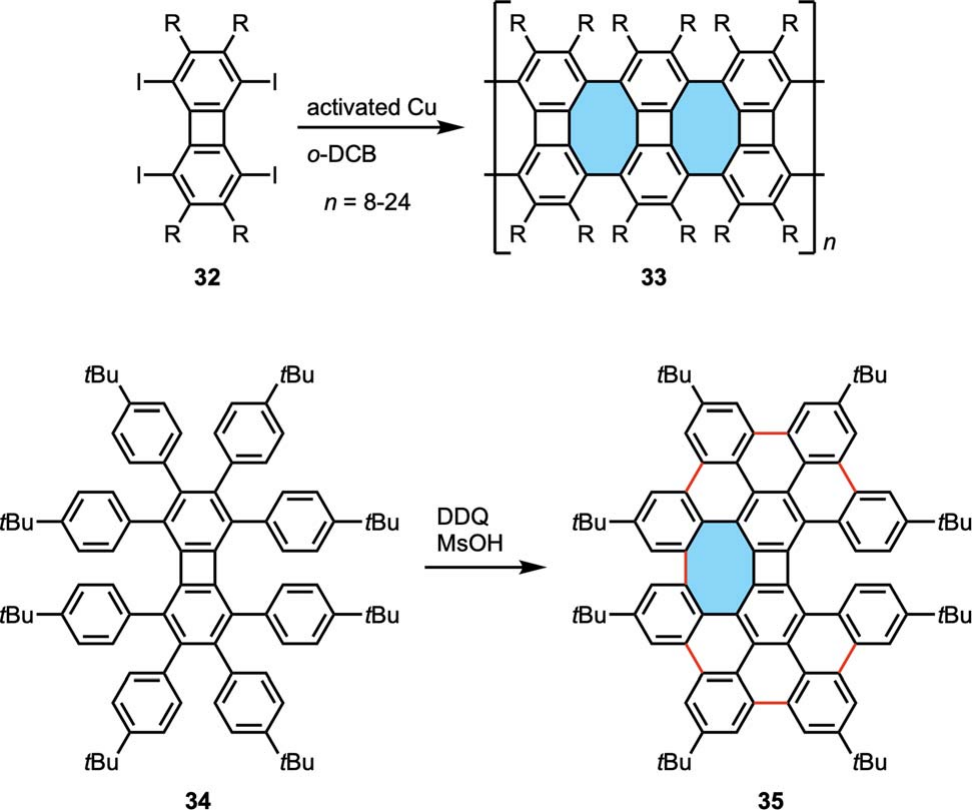
Nanoribbons 33 and expanded helicene 35 containing four-, six-, and eight-membered rings from highly substituted biphenylenes.

The group of Müllen studied the use of biphenylene-based molecules for the synthesis of graphene-like nanoribbons containing eight-membered rings.^66^ Starting from octafunctionalized biphenylenes 32, they succeeded in the copper-catalyzed polymerization reaction along the N–S axis to obtain isomeric graphene structures 33 containing eight-membered rings (Scheme 13). These planar polymers were characterized by MALDI-TOF MS, NMR and Raman spectroscopy, UV-vis absorption and emission spectroscopy. In addition, octa(*p-tert*-butylphenyl)biphenylene substrate 34 underwent intramolecular cyclodehydrogenation in the presence of DDQ and methanesulfonic acid (MsOH). Under Scholl reaction conditions, the reaction smoothly proceeded to give a nanographene molecule, the structure of which is proposed as 35 that contains one eight-membered ring.

**Scheme 14:**
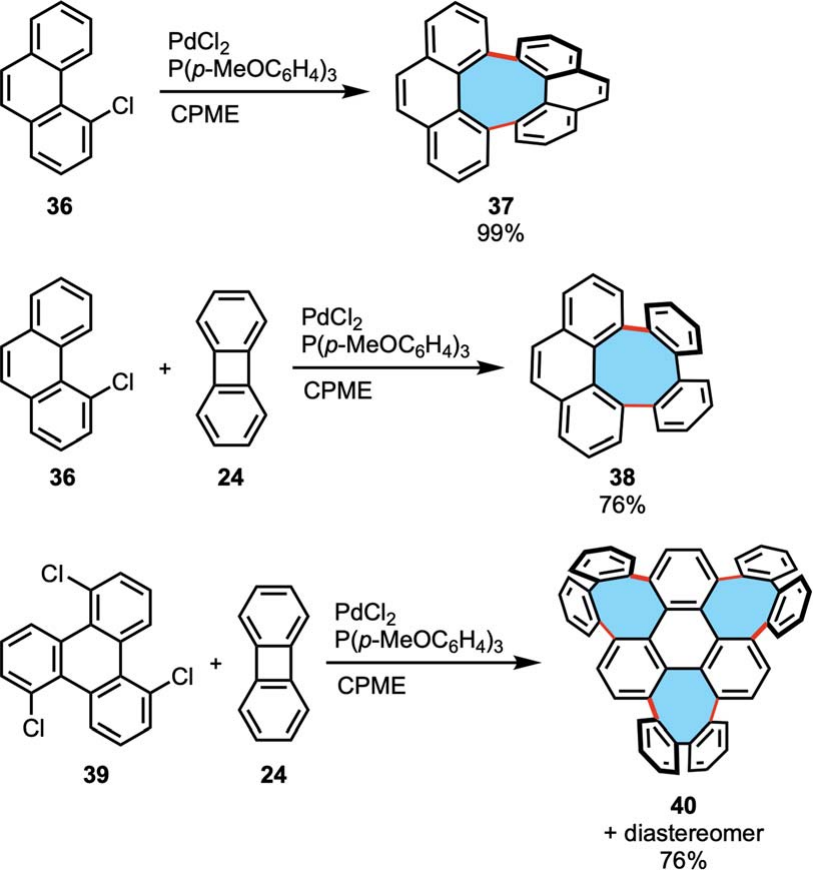
Annulative coupling of PAHs that form eight membered-rings.

Our group developed a versatile and robust method for the efficient synthesis of previously inaccessible tetraphenylenes and other eight-membered ring analogues *via* annulative coupling reactions. *Bay*-chlorinated PAHs 36 underwent annulative dimerization *via* double C–H activation in high yields. Additionally, equimolar annulative cross-coupling of chlorophenanthrene 36 with 24 in the presence of a palladium catalyst gave the corresponding octagonal products 38 with high cross-selectivity (Scheme 14).^67^ Notably, the three-fold cross-coupling reaction of a tri-chlorinated triphenylene 39 with 24 afforded the construction of 40, a novel and previously unreachable structure with three eight-membered rings and a highly curved framework.

##### 2.1.1.4. Diels–Alder cycloaddition of diynes and furans

**Scheme 15:**
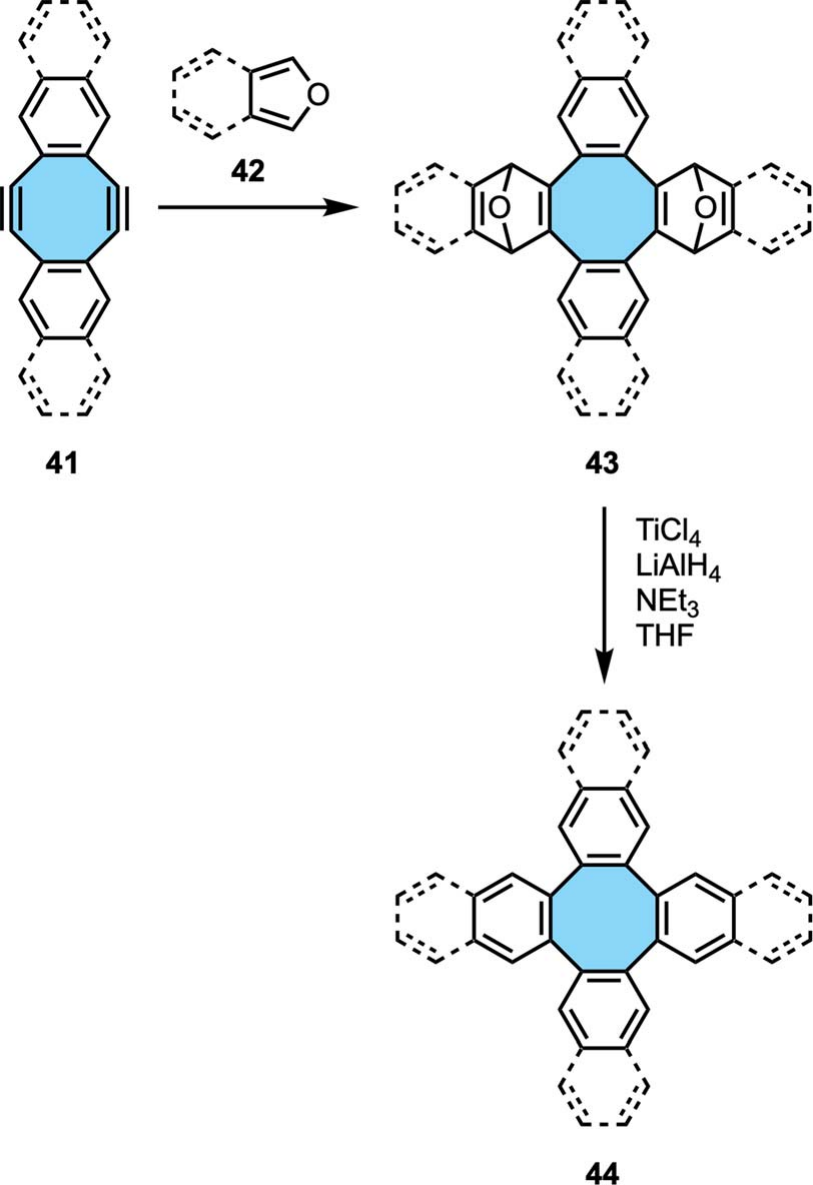
Diels–Alder cycloaddition and deoxygenation from cyclooctadienediyne.

Wong and Sondheimer pioneered the production of dibenzocyclooctadienediyne, known as Sondheimer–Wong diyne,^68,69^ a stable planar COT embedded with conjugated polycycles. Subsequently, the Diels–Alder cycloaddition of families of diacetylenes 41 and furan derivatives 42 allowed the formation of transient endoxides 43 with intrinsic curvature. Then, deoxygenation with low-valent titanium produced *in situ* by reduction of TiCl_4_ with LiAlH_4_ (ref. 70) yielded the corresponding octagon-containing compounds 44 (Scheme 15). X-ray crystal structure analyses confirmed the curved geometry rising from the central cyclooctatetraene ring.^71–73^

**Scheme 16:**
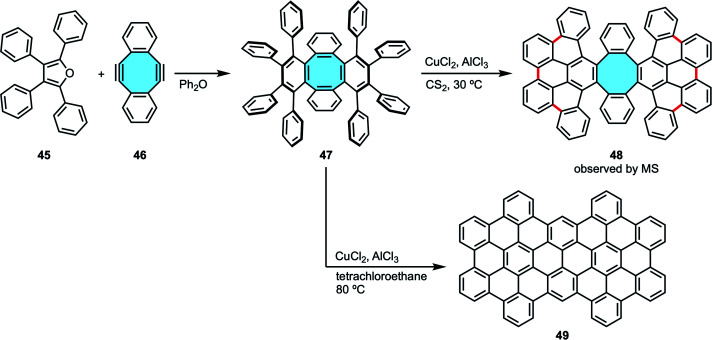
Octaphenyl-tetraphenylene 47 by Diels–Alder reaction of bisalkyne 46 and tetracyclone 45.

Ever since, the synthesis of tetraphenylene analogs by the Diels–Alder cycloaddition-deoxygenation protocol has given access to increasingly more complex nanocarbons containing π-extended eight-membered rings. With this protocol as the starting point in their study of cyclodehydrogenation/rearrangement of tetraphenylenes, Müllen and coworkers synthesized an octaphenyl-substituted tetraphenylene 47 by Diels–Alder reaction of cyclooctadienediyne 46 and tetracyclone 45 (Scheme 16).^74^ Subsequent Scholl reaction using CuCl_2_ and AlCl_3_ gave two different nanocarbons depending on the reaction conditions. At 30 °C, they observed by MS analysis nanocarbon 48 with the eight-membered hub intact that underwent formation of six new C–C bonds between the phenyl substituents. The extension of new C–C bonds formed did not reach to close the outer ring, hence the formation of an [8]circulene did not take place. If higher temperature was used, they obtained a completely fused fully-benzenoid framework 49 after ring rearrangement.

**Scheme 17:**
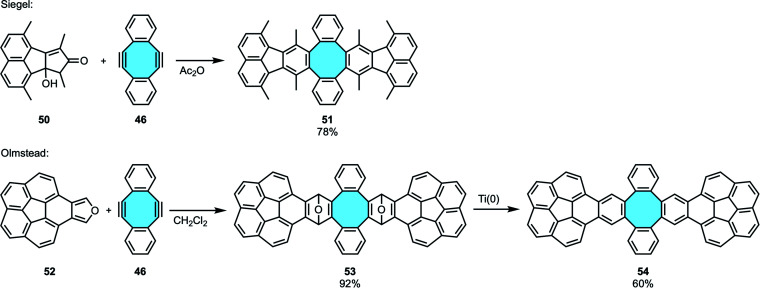
Pincer-shaped polycyclic hydrocarbons by Diels–Alder cycloaddition.

In 2005, Siegel and coworkers employed the Diels–Alder reaction protocol to synthesize nonplanar PAH 51 with negative curvature consisting of a COT hub and two fluoranthene subunits (Scheme 17).^75^ Soon after, Olmstead and coworkers obtained a larger double concave hydrocarbon with a central non-six-membered ring of similar structure (Scheme 17). The extended framework 54 in a tweezer shape was obtained after linking two corannulene derivatives 52 to the central moiety 46 featuring an eight-membered ring, followed by subsequent deoxygenation. The structure was identified by NMR analyses. In addition, they could form a stable complex of fullerene C_60_ allocated between the tweezer arms that gave single crystals suitable for X-ray diffraction analyses.^76^

**Scheme 18:**
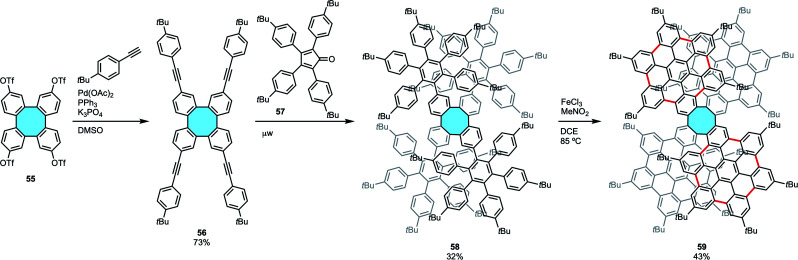
Nanographene 59 with an eight-membered hub and up to 53 fused rings.

Following a modified strategy of the Diels–Alder coupling of cyclooctadienes and furan derivatives, Martín and coworkers recently synthesized two 3D nanographenes with an eight-membered hub and up to 53 fused rings (Scheme 18).^77^ In a stepwise in–out approach, they first produced tetraphenylene 56 by means of C–H activation of the corresponding iodobiphenyl and subsequent Sonogashira coupling of 55 with 4-*tert*-butylphenylacetylene.^53^ Diels–Alder cycloaddition with cyclopentadienone 57 decorated the side wings around the eight-membered ring structure 58, and Scholl reaction afforded 24 new C–C bonds of tetraphenylene 59 functionalized with four fused hexa-*peri*-hexabenzocoronene (HBC) moieties. Nanographene 59 was characterized by MALDI-TOF, NMR, FTIR and UV-vis spectroscopies, and X-ray crystal structure analyses confirmed the saddle structure of the nanographene with planar HBCs functionalities around the eight-membered ring.

##### 2.1.1.5. [2 + 2 + 2] inter–intramolecular cycloaddition of triynes

**Scheme 19:**
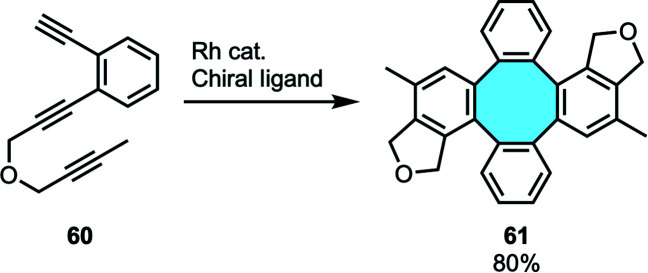
Inter- and intramolecular cycloaddition of *o*-phenylene-tethered triynes.

In 2009, Shibata and coworkers reported a rhodium-catalyzed enantioselective dimerization of triynes 60 to give chiral tetraphenylenes.^78^ Their series of publications studying the [2 + 2 + 2] inter- and intramolecular cycloaddition protocol were the first to achieve a catalytic and highly enantioselective synthesis of chiral tetraphenylenes (Scheme 19).^79,80^ This elegant protocol gives access to highly functionalized tetraphenylenes 61, although the intrinsic nature of the substrates used derives in a limited offer of accessible structures.

##### 2.1.1.6. Ring expansion

**Scheme 20:**
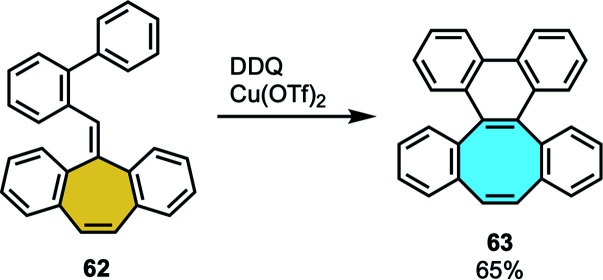
Ring expansion from seven-to eight-membered rings.

Jin and coworkers focused their efforts on the ring expansion of π-extended polycyclic arenes.^81^ Starting from *o*-biphenyl-tethered methylenecirculenes fused with a seven-membered ring 62, the methodology consisted of a sequential/tandem single-electron oxidation, intramolecular spirocyclization and 1,2-aryl migration process (Scheme 20). The X-ray crystal structure analysis of the octagon-containing product 63 featured saddle-shaped structure. In addition, the substrate scope with functionalization on varied aromatic positions allowed them to study the substituent effect in the reaction. Furthermore, they additionally functionalized the dibenzocyclooctaphenanthrenes (dbCOTPs) frameworks at the bared double bond of the eight-membered ring, demonstrating the synthetic utility of this family of products. A quinoxaline-fused dbCOTP was obtained after the formation of a diketone product and condensation with 1,3-diaminobenzene, and after a synthetic route consisting of formation of the octagon-cyclic alkyne, Diels–Alder cycloaddition and subsequent deoxygenation/aromatization, they could extend the π-system with a 1,4-diphenyl naphthalene moiety. Recently, Müllen and coworkers tried to use this protocol in the tandem oxidative ring expansion of a cyclotrimerized analog of 62, which did not afford a three-fold derivative of compound 63 consisting of three COT units attached to a central benzene ring. Instead, they used cyclotrimerization reaction to access the corresponding triangulene.^82^

##### 2.1.1.7. [4 + 4] cyclodimerization reaction of biindenyl derivatives

**Scheme 21:**
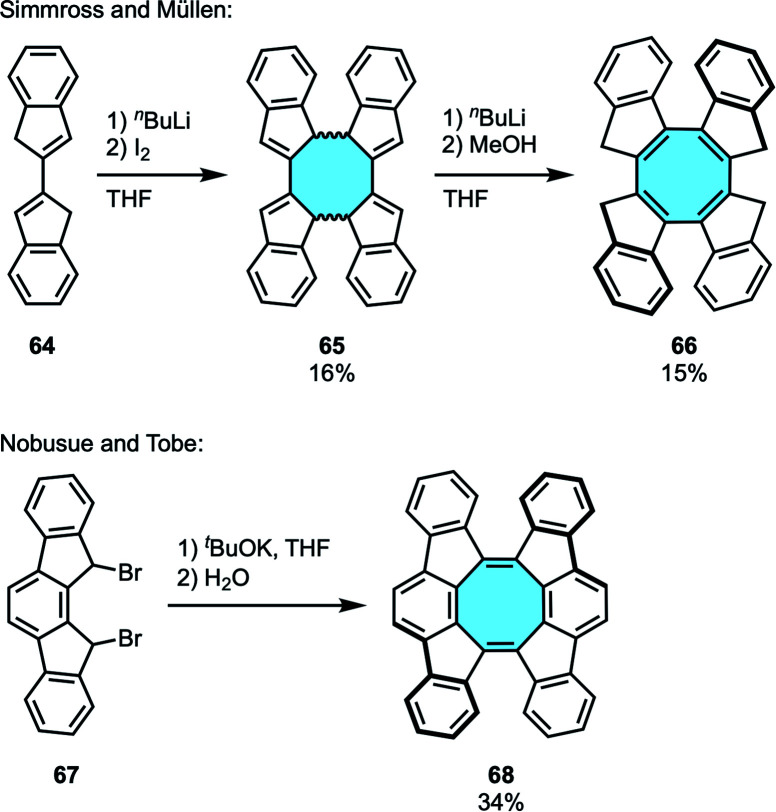
[4 + 4] cyclodimerization towards cyclooctatetraene derivatives.

In 1993, Simmross and Müllen reported the synthesis of the eight-membered ring 66*via* [4 + 4] cyclodimerization employing biindenyl 64 as the starting material (Scheme 21a).^83^ The benzo-fused dicyclopentadiene 64 underwent oxidative coupling to give the cyclic dimer 65, with subsequent deprotonation-protonation to yield twisted tetraindeno-fused cyclooctatetraene 66 in 15% yield. This work inspired Nobusue and Tobe in the stepwise [4 + 4] cyclodimerization and elimination protocol for the synthesis of a saddle-shaped PAH with a central cyclooctatetraene ring fused by two indenofluorene moieties 68*via* an *in situ* formed indenofluorene derivative (Scheme 21b).^84^ X-ray crystal structure analysis confirmed the twisted conformation of 68 that averts intramolecular steric repulsions of the side wing moieties.

##### 2.1.1.8. Cyclization or annulation of linear precursors

Last, ring closure methodologies might seem a very straightforward method to obtain fully conjugated twisted eight-membered rings, but this synthetic strategy remains an elusive challenge. These entropically disfavoured processes suffer from transannular interactions that lead to intermolecular oligomerization or polymerization byproducts instead of the desired intramolecular cyclization target molecules. There are however brilliant examples to highlight in the synthesis of PAHs with an eight-membered ring from linear substrates: thiazole tetramer *via* head-to-tail connection by C–H arylation,^85^ or selected examples of partially conjugated cyclooctene derivatives by cyclooctene-carbopalladation of allenes,^86^ [5 + 2 + 1] cycloadditions of ene-vinylcyclopropanes,^87^ [4 + 4 + 2] cycloaddition/alkyne insertion of dieneynes,^88^ transition-metal-catalyzed cyclizations of aryl ethers,^89^ ring-closing metathesis,^90^ and by transition-metal-catalyzed hydroarylations of alkynes.^91^ Recently, the group of de Bruin reported the metalloradical synthesis of partially conjugated cyclooctene derivatives.^92,93^

#### 2.1.2. Protocols towards stitching around the eight-membered central ring

##### 2.1.2.1. Intramolecular Pd-catalyzed benzannulation of tetraphenylenes

**Scheme 22:**
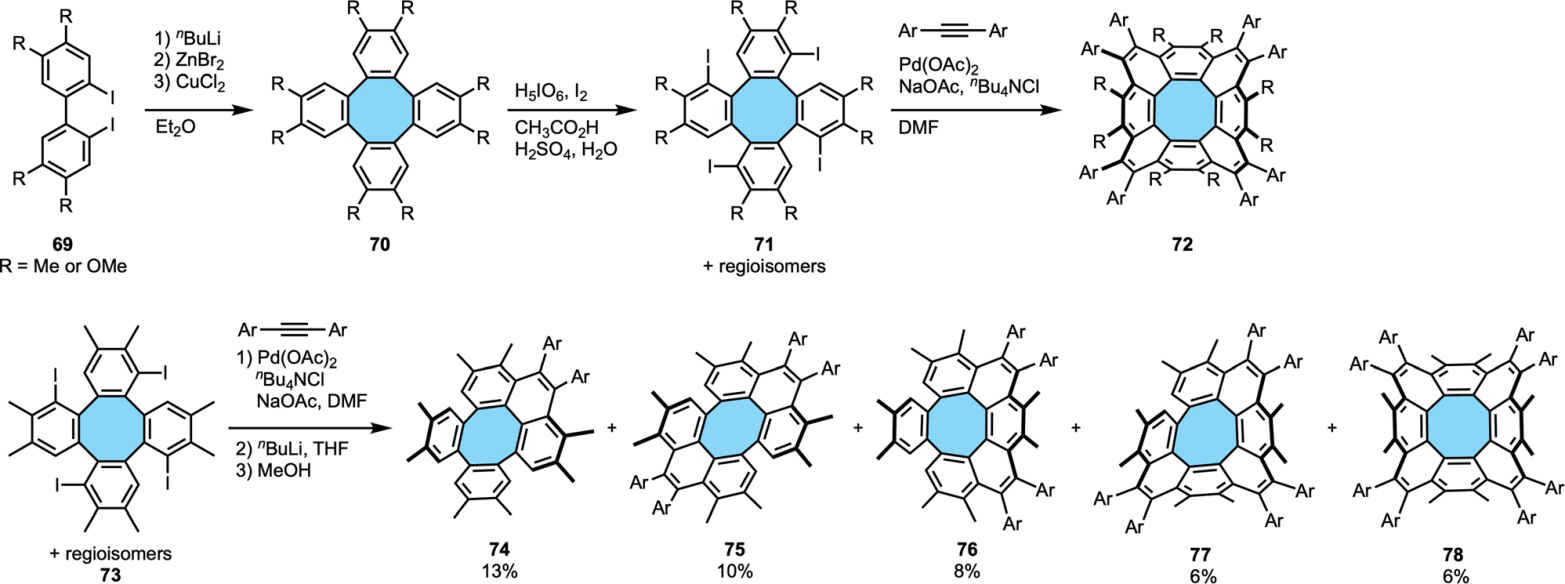
[8]Circulenes *via* Pd-catalyzed annulation of tetraiodo-substituted tetraphenylenes with diarylethynes.

In 2013, Wu and coworkers pioneered the synthesis and structural elucidation of [8]circulenes.^94^ The reported approach consisted of the four-fold intermolecular Pd-catalyzed annulation of tetraiodo-tetraphenylenes 71 with symmetrical diarylethynes (Scheme 22a). Following Iyoda's protocol for the synthesis of substituted tetraphenylenes 70 by Cu(ii)-mediated cyclodimerization of diiodobiaryls 69,^45^ they prepared the substrates by subsequent iodination. The latter step gave regioisomeric mixtures of four regioisomers of 71 with one iodo substituent in each *bay*-region. Then, the palladium-catalyzed annulation process fused each *bay*-region in the periphery of the central eight-membered ring to yield the corresponding *peri*-substituted [8]circulene derivatives 72. Unfortunately, in accordance with predictions by Clar's theory of aromaticity, these compounds were only stable within a range of one to few days in solution. The structure of the peribenzenoid-substituted [8]circulenes 72 was characterized by NMR spectroscopy and X-ray crystal structure analysis. The former suggested that the [8]circulenyl scaffold was not planar, while crystallography analyses revealed that the framework has a saddle-shaped structure. In addition, only one conformer of compound 72 was observed in the crystal structure analysis.

In their next research work, Wu and coworkers could shed light on the ring flipping mechanism of [8]circulenes. In a clever synthetic approach, they could isolate and characterize the products yielded by successive benzannulations of tetraiodo-tetraphenylenes 73 – *i.e.*, monoannulated 74, *para*-diannulated 75, *ortho*-diannulated 76, triannulated 77 and periannulated 78 – during the one-pot concerted Pd-catalyzed C–H activation and alkyne annulation (Scheme 22b).^95^ They could then observe that consecutive annulations introduce strain gradually, changing the molecular geometries and physical properties of the products. Their stability was in the range of the previous study. For 78, the solution was only stable for up to 2 weeks at room temperature. With these dynamic behavior results in hand, they suggested that instead of an energetically demanding ring inversion mechanism, the flipping of [8]circulenes may proceed through pseudorotation.

##### 2.1.2.2. Scholl reaction

**Scheme 23:**
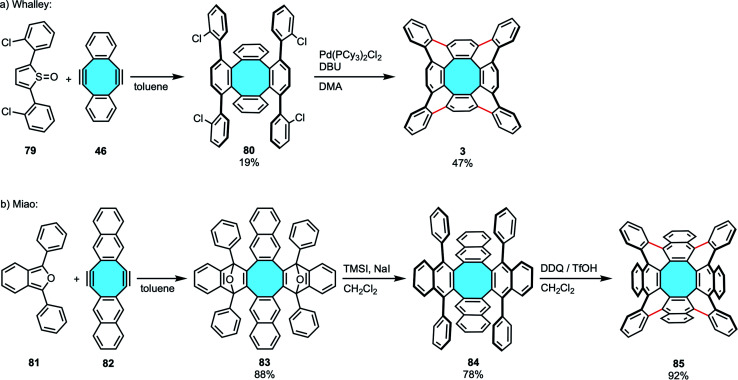
Synthesis of tetrabenzo[8]circulene 2 (a) and octabenzo[8]circulene 85 (b) *via* two-fold Diels–Alder cycloaddition and Pd-catalyzed arylation/Scholl reaction.

In 2013, Sakamoto and Suzuki synthesized for the first time the fully benzenoid tetrabenzo[8]circulene 2 that is much more stable than [8]circulene (1), albeit in an alternative out–in cyclization that will be described in the next section (*vide infra*, Scheme 25b).^96^ One year later, the group of Whalley designed the first in–out synthetic route for the preparation of 2 (Scheme 23a).^97^ First, they employed 46 that underwent a two-fold Diels–Alder cycloaddition. Commonly used substrates such as 2,5-diphenylfuran or 2,5-diphenylthiophene dioxide did not yield the desired tetraphenylene derivatives, likely as the reactions took place at higher temperatures and the product decomposed. In contrast, 2,5-diarylthiophene-1-oxides 79 were found to be the most reactive substrates. After forming the corresponding tetraphenylene derivative 80, subsequent Pd-catalyzed intramolecular C–H arylation reaction closed the framework to yield the exceptionally stable tetrabenzo[8]circulene 2, which did not show any decomposition after months of storage at room temperature. NMR and X-ray crystal structure analyses confirmed the 3D structure of the fully benzenoid framework. In a follow-up work, the same group reported an improved procedure that afforded scaffolds decorated with both electron-rich and electron-poor functional groups in higher yields.^98^ The alternative synthetic route differed in the second step where, instead of a Pd-catalyzed arylation, they made use of TfOH and DDQ to oxidatively cyclodehydrogenate the tetraphenylene transient products under Scholl reaction conditions.

In 2019, Miao and coworkers expanded Whalley's outwards strategy to form octabenzo[8]circulene derivatives. To achieve the synthesis of fully *ortho*-benzannulated [8]circulene 85, the first step was the two-fold Diels–Alder reaction of 1,3-diarylbenzofurans 81 with naphthalene-containing diyne 82.^99^ Cleavage of the oxygen bridges of 83 and aromatization were followed by a Scholl reaction with DDQ and TfOH to afford the saddle-shaped polycyclic backbone 85 (Scheme 23b). In comparison to Whalley's results, the higher yields observed with this procedure were allegedly attributed to the naphthalene moieties when compared to that of benzene subunits.

**Scheme 24:**
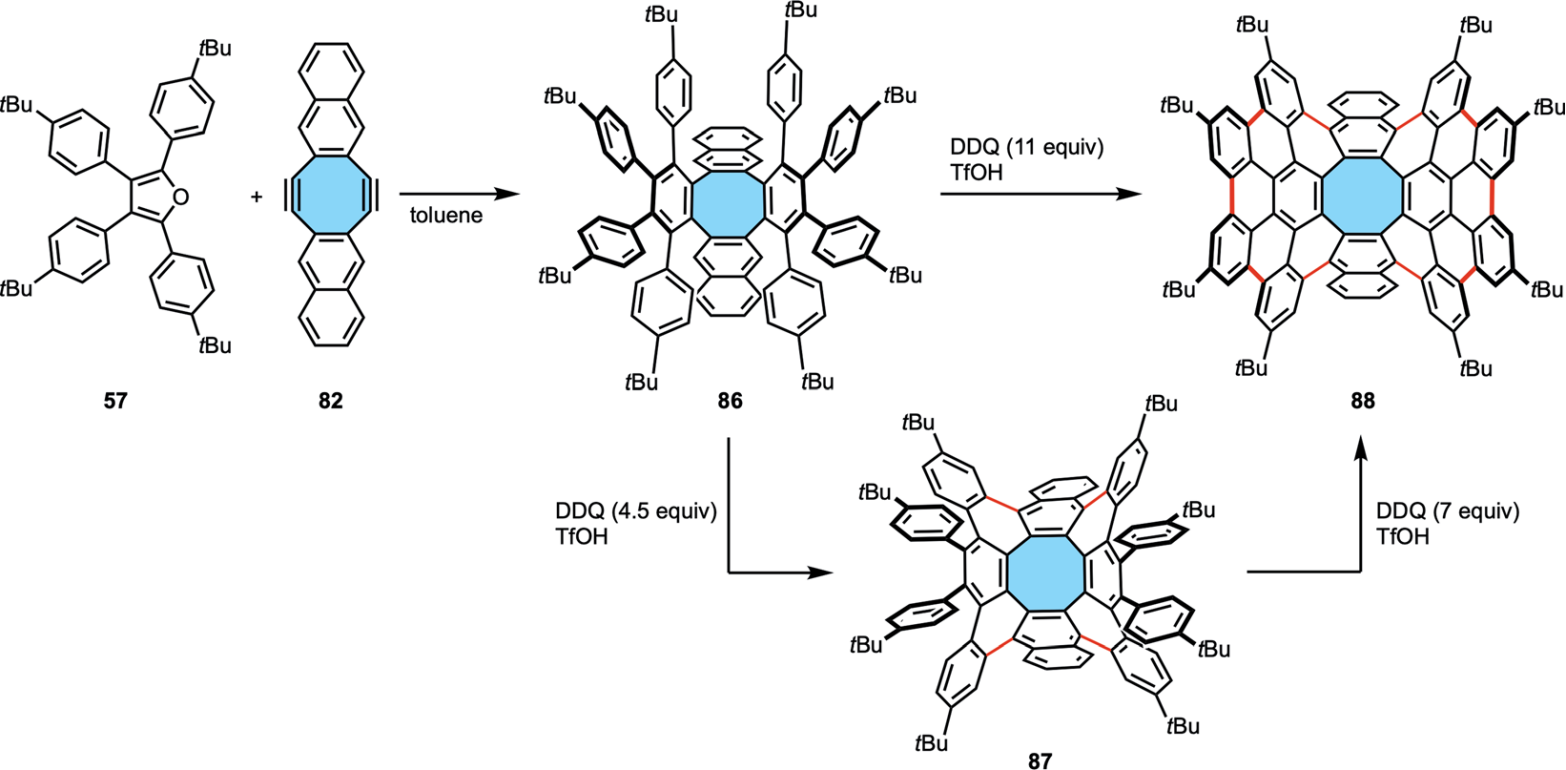
Synthesis of an [8]circulene derivative 88 containing 80 sp^2^ carbons by reaction of naphthannulated cyclooctadiyne 82 with cyclopentadienone 57*via* two-fold Diels–Alder cycloaddition and Scholl reaction.

The robustness of this method was further confirmed by Miao and coworkers with the synthesis of an octagon-embedded aromatic saddle consisting of 80 sp^2^ carbons (Scheme 24).^100^ The formation of ten new aryl–aryl bonds in making 88 was achieved in a straightforward manner using 11 equivalents of DDQ, but a stepwise formation is also possible. This alternative employed 4.5 equivalents of DDQ to form first the four new C–C bonds needed to create the [8]circulene core of 87, and subsequent use of 7 equivalents of oxidant closed the framework with six additional new C–C bonds with comparable yield results to the single-step reaction. This contrasts with the report by Müllen and coworkers (*vide supra*, Scheme 16), where they could only partially cyclize compound 48 along with the external aryl rings and suffered from skeletal rearrangements under Scholl reaction using harsher conditions.^74^

**Scheme 25:**
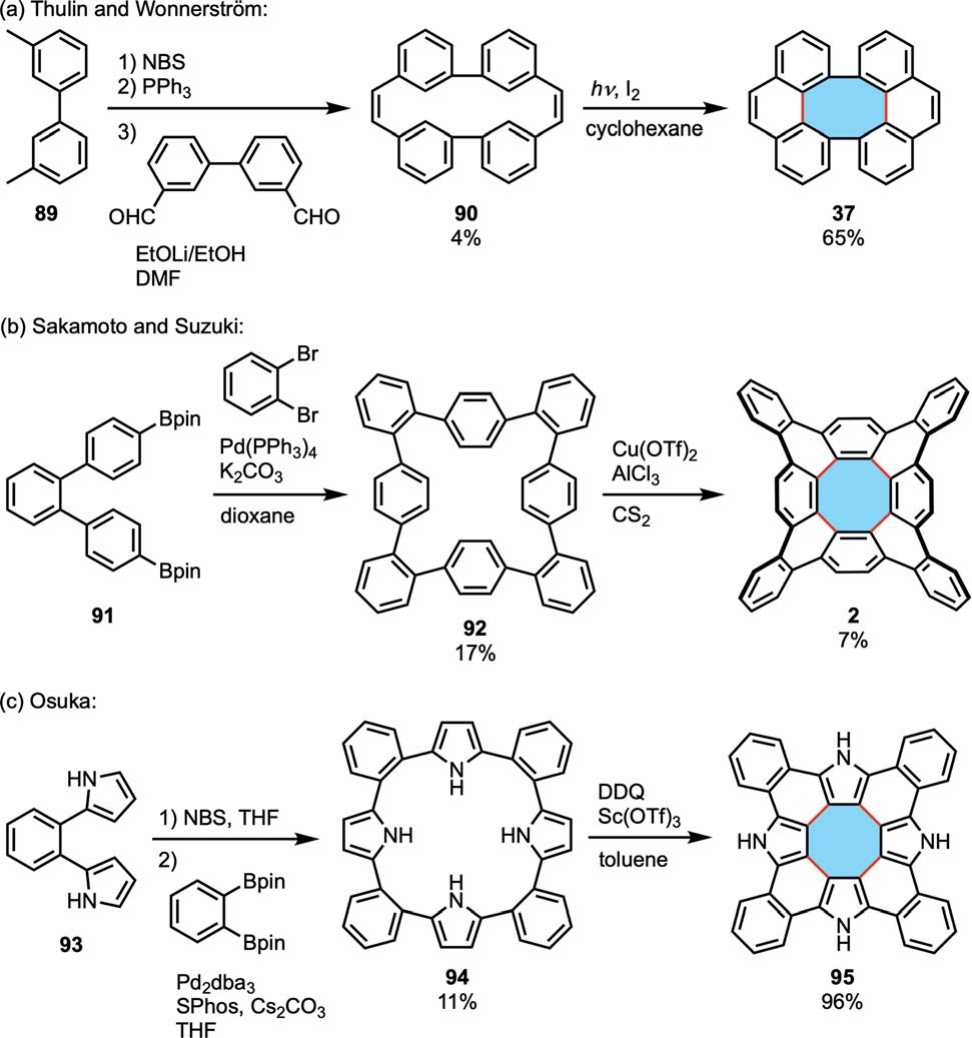
Synthetic strategies for (a) dibenzotetraphenylene 37, (b) tetrabenzo[8]circulene 2 and (c) tetrabenzotetraaza[8]circulene 95 by fold-in approaches.

### 2.2. Out–in cyclization strategy

**Fig. 5:**
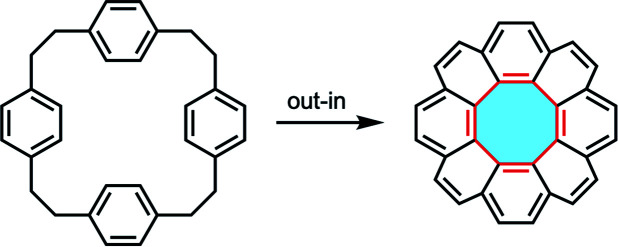
Schematic illustration of the out–in cyclization approach towards [8]circulene derivatives.

Another method for the construction of [8]circulene derivatives is the out–in approach. This method begins with the syntheses of macrocyclic precursors that subsequently undergo intramolecular ring-closing reactions (Fig. 5). As the central eight-membered hub is constructed, the nanographene folds in to accommodate the appropriate Gaussian curvature of the fully-fused product. Stępień called this strategy the fold-in approach and demonstrated the practicality in the synthesis of bowl-shaped compounds.^101,102^

As an early example of the fold-in synthesis to form an eight-membered ring, although unsuccessful in the synthesis of [8]circulene,^19^ Thulin and Wennerström reported the synthesis of dibenzotetraphenylene 37.^103^ Cyclic diene precursor 90 was prepared in three steps *via* a Wittig reaction and treated with iodine under photoirradiation to afford the saddle-shaped product (Scheme 25a). Although they provided the viability of the fold-in synthesis to construct an eight-membered ring, this strategy required the synthesis of a well-designed precursor. Therefore, the fold-in method was not applied to the synthesis of [8]circulenes for 35 years since their report.

In 2013, Sakamoto and Suzuki reported the synthesis of tetrabenzo[8]circulene 2.^96^ They designed an octaphenylene precursor 92 to construct the saddle-shaped framework following the fold-in approach, reporting the second synthesis of [8]circulenes after Wu achieved it with the alternating in–out method (*vide supra*, Scheme 22a). The macrocyclic precursor was prepared using Suzuki–Miyaura coupling and treated with Cu(OTf)_2_ and AlCl_3_ to afford tetrabenzo[8]circulene in 7% yield (Scheme 25b).

Tanaka, Osuka, and coworkers made use of the fold-in approach to achieve the synthesis of tetrabenzotetraaza[8]circulene 95 in 2015.^104^ The tetrapyrrolic precursor 94 was designed in advance and oxidized with DDQ and Sc(OTf)_3_ to give 95 in 96% yield (Scheme 25c). Although the heteronanographene has a large planar structure, it was soluble in THF and DMSO. Judging from the result of X-ray diffraction analysis, hydrogen-bonding interactions likely affect the packing structure and provide high solubility.

**Scheme 26:**
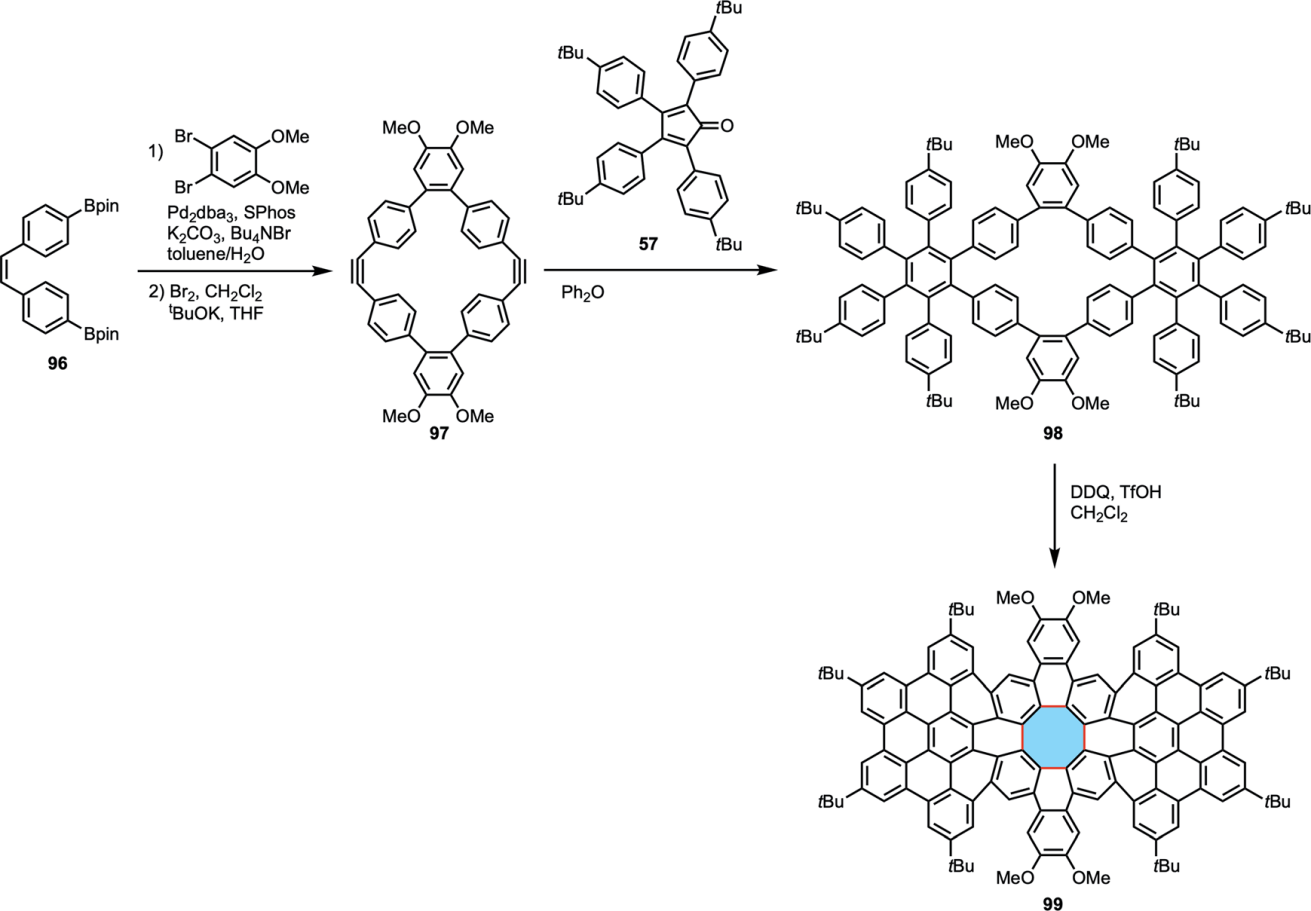
Synthesis of [8]circulene derivatives 99 consisting of 96 sp^2^ carbon atoms.

Miao and coworkers synthesized [8]circulene derivatives consisting of 96 sp^2^ carbon atoms.^105^ An elegant stepwise protocol gave access to precursor 97, a macrocyclic diyne that underwent Diels–Alder reaction with a tetracyclone derivative 57 to afford the transient product 98 bearing hexaarylbenzene moieties. Finally, Scholl reaction with DDQ and TfOH resulted in the synthesis of [8]circulene derivative 99 with formation of a polycyclic twisted framework with a central eight-membered ring and hexabenzocoronene moieties (Scheme 26). This work demonstrated the applicability of the fold-in approach to large molecular nanocarbons containing an eight-membered ring.

#### 2.2.1. Stone–Thrower–Wales rearrangements upon Scholl reactions

**Scheme 27:**
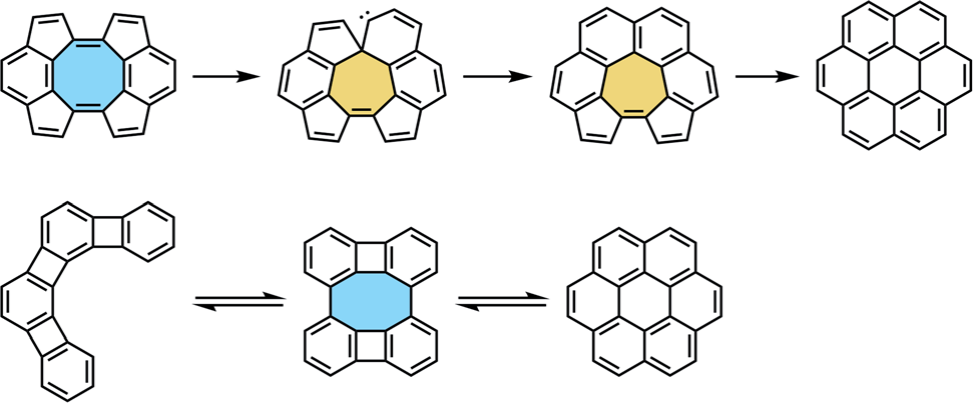
Stone–Thrower–Wales rearrangement of eight-membered rings.

Many of the examples mentioned above show the versatility and critical importance of the Scholl reaction in the intramolecular synthesis of extended fused polycyclic frameworks. Although it enables the one-pot formation of new multiple carbon–carbon bonds *via* oxidative cyclodehydrogenations in an effective manner,^106,107^ bond–switch processes can lead to skeletal rearrangements. After the first descriptions of such isomerization in graphite by Thrower,^108^ and in fullerenes by Stone and Wales,^109^ the so-called Stone–Thrower–Wales rearrangements have been observed in processes involving eight-membered rings (Scheme 27).^110,111^ The intrinsic curvature of PAHs induces strain in the fused product, and rearrangement reactions can take place, leading to unwanted products under Scholl reaction conditions. These consist of the transposition of two sp^2^-hybridized carbon atoms within a structure of other sp^2^ atoms, and computational studies suggest that radical promoters might be involved.^112^

**Scheme 28:**
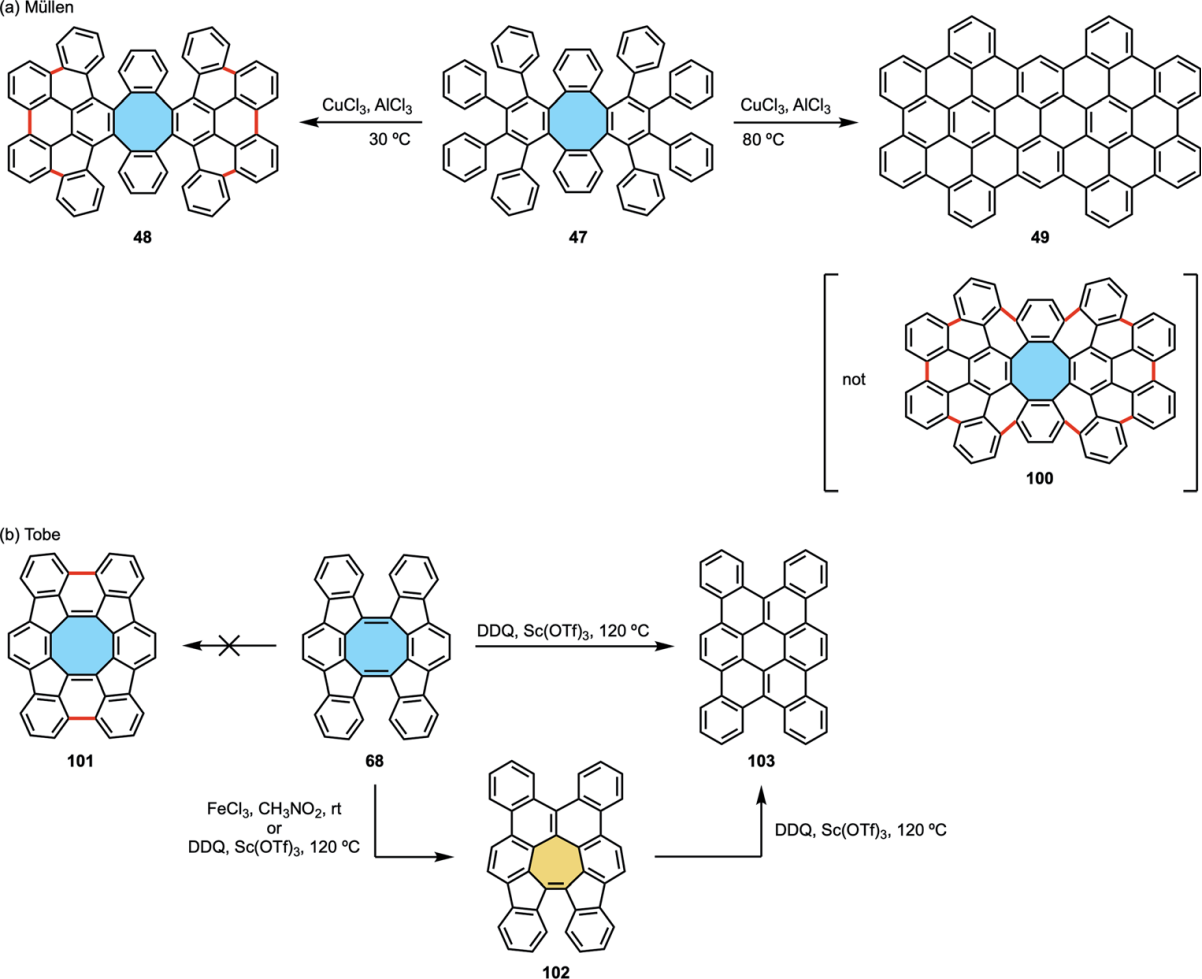
Skeletal rearrangement examples of eight-membered rings (a) to fully-fused six-, and (b) to seven- and six-membered rings.

In their synthesis of octaphenyl-tetraphenylene by Diels–Alder reaction of bisalkyne 46 and tetracyclone 45 (*vide supra*, Scheme 16), Müllen and coworkers could only cyclize partially compound 47 along the external aryl rings creating 6 new carbon–carbon bonds under Scholl conditions to yield 48. When they attempted Scholl reaction under harsher conditions to achieve the fully fused octagon-containing product with 10 new C–C bonds 100, skeletal rearrangements led to a fully-fused, fully-benzenoid product 49 (Scheme 28a).^74^ As stated above, Miao and coworkers overcame the isomerization issue by introducing the naphthalene subunits into their compound.^99^

After their [4 + 4] cyclodimerization towards twisted arene 68 comprising a cyclooctatetraene fused with two indenofluorene side wings (*vide supra*, Scheme 21b),^84^ Tobe and coworkers reported an unexpected rearrangement under Scholl reaction conditions.^113^ Instead of a fully fused product with a central eight-membered core 101, they obtained the polycyclic aromatic compound 102 bearing a seven-membered ring in the presence of FeCl_3_/CH_3_NO_2_ at room temperature or DDQ/Sc(OTf)_3_ at 80 °C. Upon treatment with DDQ/Sc(OTf)_3_ at 120 °C, either the initial cyclooctatetraene 68 or the seven-membered ring compound 102 yielded a fully-benzenoid compound 103, showing that a stepwise Stone–Thrower–Wales rearrangement might be involved (Scheme 28b).

## 3 Towards extended sp^2^ macrostructures with eight-membered rings: nanobelts, nanotubes, and porphyrin sheets

**Scheme 29:**
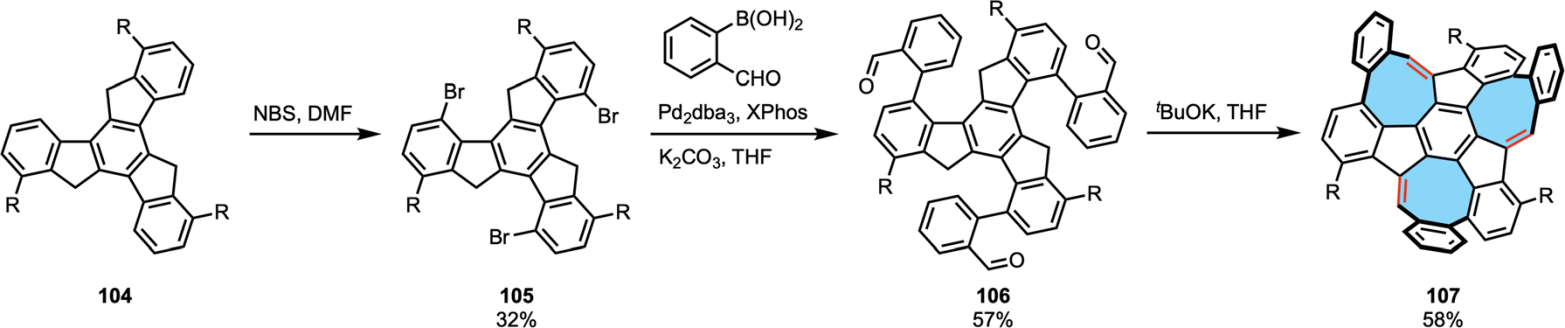
Three-step synthetic strategy towards monkey saddle-shaped nanographene 107.

The strategies summarized above give bottom-up access to a plethora of more complex polycyclic macrostructures with intriguing 3D frameworks. Mastalerz and coworkers chose a different approach in the synthesis of potential schwarzite building blocks. In 2020, instead of growing tetraphenylenes or [8]circulenes, they reported the first synthesis and characterization of a chiral monkey-saddle-shaped framework consisting of three eight-, three five- and seven six-membered rings 107, as well as its triaza analog.^114,115^ The insertion of multiple different ring sizes causes odd warping in nanographenes that greatly modifies solubility, as well as the electronic and optical properties of the material.^116,117^ In addition, these grossly warped frameworks could become the building blocks of 3D π-surface macrostructures such as fully conjugated cages or carbon schwarzites, composed of embedded five-to eight-membered rings. The final product was obtained in a three steps procedure. Substrate truxene 104 underwent three-fold bromination in the cove regions, followed by three-fold Suzuki–Miyaura coupling with 2-formylphenylboronic acid. Subsequently, the three eight-membered rings were closed in basic conditions (Scheme 29). Interestingly, Mastalerz and coworkers were also the first ones to separate the two enantiomers, later studied by CD spectroscopy. X-ray diffraction study confirmed the monkey saddle structure and no clear π-stacking was observed, in agreement with the high solubility of the product. Campaña and coworkers have recently pushed the state-of-the art in the synthesis of extended tetraphenylenes by means of modifying the Diels–Alder cycloaddition protocol. Starting from dibenzocyclooctyne instead of dibenzocyclooctadienediyne, they accessed to non-symmetric curved PAHs that do not feature the eight-membered ring in the central position. The authors performed the chiral resolution and assigned the enantiomers of the studied the doubly distorted motif and its analogs.^118^

**Scheme 30:**
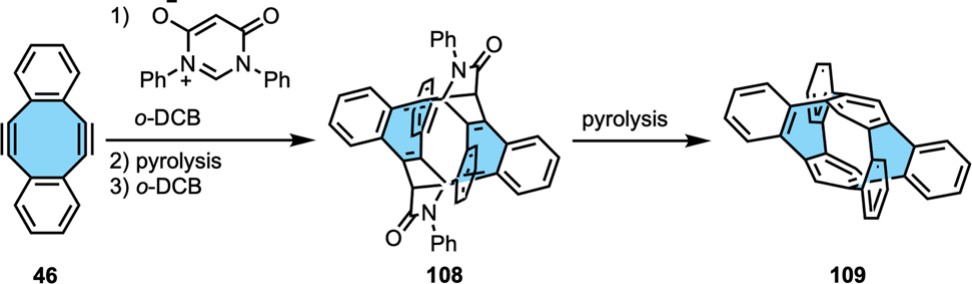
Cyclophane 109 (tetrabenzannelated 1,2,4,5-cyclophane) by dimerization reaction.

Carbon nanorings and nanobelts are other carbon allotropes that can benefit from tube-shaped eight-membered rings, since these cylindrical structures require building blocks that either are flexible or feature curvature to provide structural torsion in the structure.^119^ Wudl and coworkers imagined an elegant retrosynthetic analysis for the first synthesis of a benzannelated [2_*n*_]cyclophane with more than two benzo bridges 109. In this case, they started from dibenzocyclooctadiene-diynes 46 that would afford the novel benzo bridges in the final product [2_4_]cyclophane (Scheme 30).^120^ The eight-membered diyne underwent two-fold Diels–Alder addition, followed by pyrolysis and thermal extrusion of phenyl isocyanate to obtain a tetraphenylene derivative. Finally, dimerization and pyrolysis afforded cyclophane 109, characterized by NMR and X-ray crystal structure analyses that clearly displayed the saddle shape of each cyclooctatetraene unit.

**Scheme 31:**
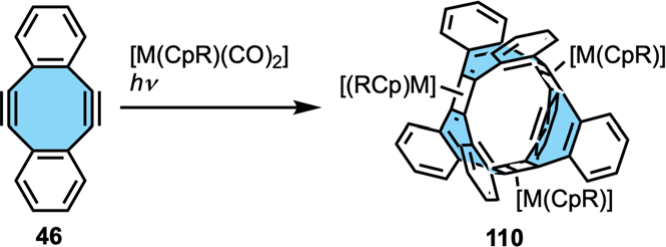
Cyclacenes by intramolecular ring closure.

In many cases, graphene nanoribbons tend to show low stability. One strategy to overcome this limitation present in rings composed of annulated benzene rings is to replace some planar rings by other moieties with boat conformation. For this reason, carbon-based nanostructures with negative curvatures that enhance the flexibility of the carbon skeleton are an interesting approach towards synthesis of belt-like systems. Gleiter and coworkers reported the first beltenes consisting of annulated conjugated four- and eight-membered rings.^121^ Irradiation of cyclooctadiyne 46 at 254 nm in the presence of different equivalents of cobalt complexes [Co(CpR)(CO)_2_] afforded four new [4.8]_3_cyclacenes 110 (Scheme 31). Mechanistic studies suggested a stepwise annulation of successive molecules of starting materials towards ring closure. X-ray crystal structure analyses confirmed the boat conformation of the eight-membered moieties with their benzene rings twisted out of the cyclobutadiene plane. In 2009, they extended the scope to rhodium derivatives, with all [4.8]_3_cyclacene derivatives showing a conjugated belt-like shape.^122^

**Scheme 32:**
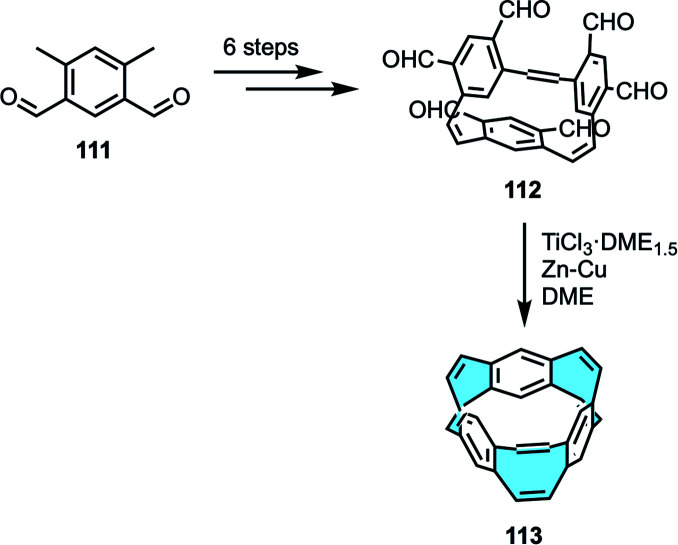
Synthesis of [6.8]_3_cyclacenes *via* ring closure.

Alternatively, Gleiter and coworkers explored the conjunction of eight- and six-membered rings to yield [6.8]_3_cyclacenes 113, the first example of purely hydrocarbon cyclacenes.^123,124^ While the synthesis of the four-membered ring containing belts was one-pot, the ring closure of the benzene-octagon derivatives was obtained in a stepwise approach (Scheme 32).

**Scheme 33:**
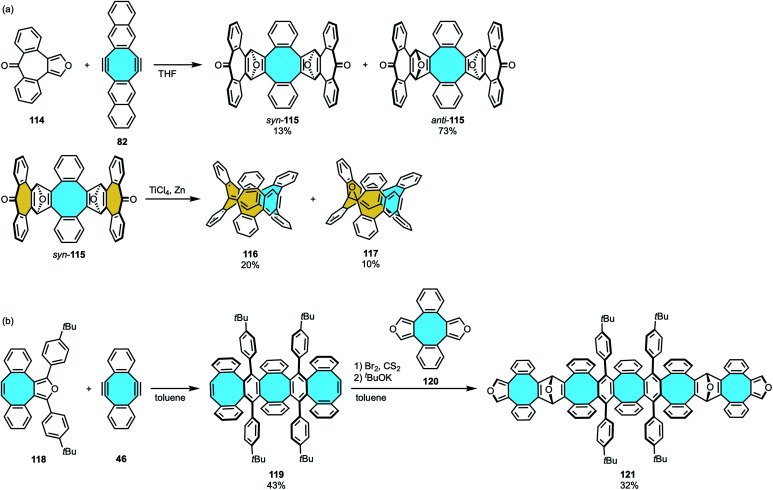
(a) Two-step synthesis of tetraphenylene-containing nanoring 116; (b) synthetic attempts towards carbon nanobelts by sequential Diels–Alder cycloaddition.

The use of consecutive rings with inherent curvature to afford nanorings was as well the strategy followed by Miao and coworkers, inspired by previous approaches in the outward synthesis of pincer-shaped [8]circulene derivatives.^72,73,97–99^ The two-fold Diels–Alder cycloaddition of naphthannulated cyclooctadiyne 82 and a heptagon-containing furan 114 gave *syn*- and *anti*-addition products with seven- and an eight-membered rings along with the transient formation of bent endoepoxides (*syn*-115 and *anti*-115, respectively) (Scheme 33a).^125^ The *syn*-product with pincer-shaped geometry was isolated and utilized in the McMurry reaction in the presence of TiCl_4_/Zn to finally yield nanoring 116 that featured two seven- and one eight-membered rings.

Further attempts by Chen and Miao to expand the stepwise Diels–Alder procedure to synthesize a fully-fused negatively-curved macrocyclic nanobelt did not give the target molecule. The two-fold Diels–Alder cycloaddition of Sondheimer–Wong diyne 46 with an octagon-containing cyclopentadienone 118 followed by *in situ* decarbonylation gave a nanographene featuring three eight-membered rings 119. In the second step, *in situ* formation of the dialkyne adduct and treatment with cyclopentacycloocten-2-one 120 was expected to yield a nanobelt of alternatingly fused six- and eight-membered rings (Scheme 33b).^126^ Instead, the additional two-fold Diels–Alder cycloaddition gave a pincer-shaped nanographene 121.

**Scheme 34:**
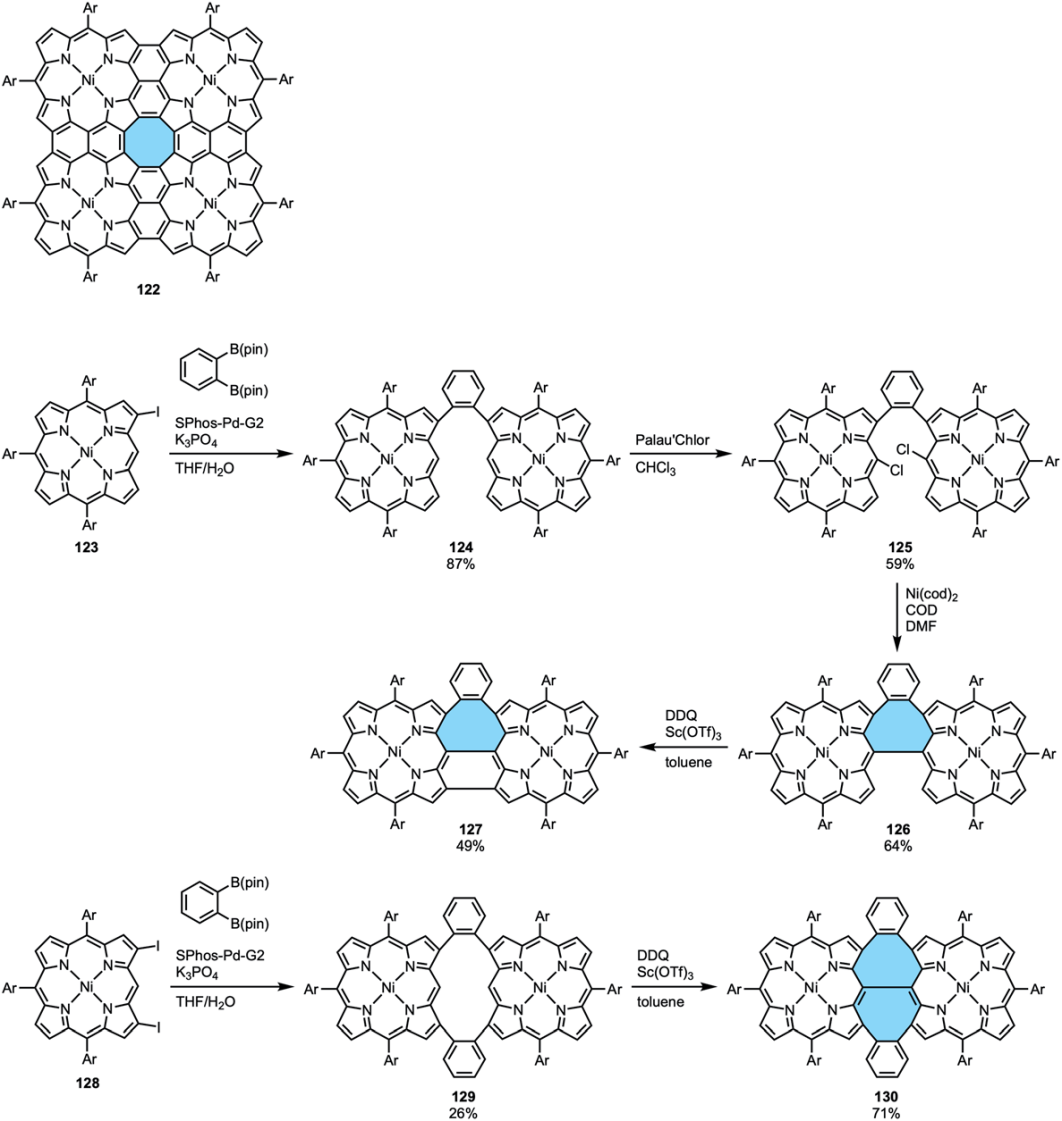
Eight-membered ring-containing curved porphyrin sheets.

Porphyrin tapes are an intriguing kind of linear arrays of PAHs that can have their properties tuned by embedding different heteroatoms or metals within their frameworks.^127^ When porphyrin tapes are extended in tetrameric form, the so-called porphyrin sheets 122 are formed (Scheme 34a). In both cases, they consist of fully conjugated networks with highly planar structures, highlighting that tetrabenzotetraaza[8]circulene 95 are the central motif of porphyrin sheets 122.^128,129^ In 2018, Fukui and Osuka reported a new member in the family of porphyrin-based macrostructures that displays structural curvature, the porphyrin arch-tapes. They followed an out–in approach from 2-iodo- and 2,18-diiodo-Ni^II^ porphyrin (123 and 128) to synthesize porphyrin arch-tapes 127 and 130 containing one or two eight-membered rings, respectively (Scheme 34b).^130^ The contorted structures were determined by X-ray crystal structure analyses, and DFT calculations showed that the more saddle-shaped the structure is, the electrochemical HOMO–LUMO gap becomes smaller.

## 4 Summary and outlook

To date, efforts in the synthesis of negatively curved carbon schwarzites and other sp^2^-carbon allotropes with mixed Gaussian curvature have been unsuccessful. One of the potential gates to access these challenging 3D frameworks might be the bottom-up approach, and in this review, we have summarized the main highlights and progress in the synthesis of saddle-shaped curved polycyclic structures bearing eight-membered rings.

The distortion introduced in carbon lattices by the presence of non-benzenoid rings – *e.g.* not only distancing them from planarity and forcing the warped π-backbones to retain the typical C–C bond distances, but also causing changes in connectivity – is significantly depending on the degree of contortion, and it grants these structures with unique physical and chemical properties when compared with planar analogs, such as greater solubility and flexibility, as well as higher fluorescence with larger HOMO–LUMO gaps, and bathochromic shifts in the absorption spectra.^66,77,99,105,115–117,131^ These features have led to the current trend among many research groups, as highlighted in this review, where there has been a great increase in research publications that show new synthetic approaches to further advance in more challenging targets.

We envision new and innovative synthetic discoveries that enrich the field in the years to come, focusing in finding further applications for topographical curvatures in the preparation of optoelectronic devices, such as organic field-effect transistors (OFETs), organic light-emitting diodes (OLEDs), or organic photovoltaics (OPVs), as well as photovoltaics and sensors.

The directed and controlled synthesis of more complex octagon-containing negatively curved lattices will open new possibilities that will join those already existing that use other non-benzenoid rings, with special highlight on chiral PAHs and other tailor-made warped structures with chiroptical properties. In addition, combining the know-how on planar and non-planar networks, merging five-, seven-, eight-membered rings and beyond, could give way to prepare unprecedented and sophisticated π-conjugated nanocarbons, such as carbon nanobelts and nanocages, schwarzites (the aforementioned Mackay–Terrones crystals), fullerene dimers, toroidal carbon nanotubes with negative or mixed Gaussian curvatures.

As this research field matures and synthetic protocols establish with general and efficient large-scale strategies towards the commercial availability of curved nanocarbons, reports of larger and more exotic lattices should leave some room in the literature to adjacent studies. Examples of prospective research projects would address the fundamental understanding towards more rational structure–property relationships, such as mechanistic investigations that unravel the role of structural dynamics in the chemical processes as well as the properties and performance of the curved topographies obtained in new and complex structures. New reports will appear in the literature with comprehensive evaluation of final-stage production, underlining the scientific and engineering novelty the materials discovered, and considering the life cycle assessments that examine the environmental impact of those technologies.

The strategies shown here would offer a fine inventory on the controlled synthesis of nanostructures that many researchers will likely employ as an introduction to the field or as an update in the state-of-the art of this exciting field.

## Author contributions

K. I. and K. M. conceived the idea of the review and supervised the work. G. G. M. designed the structure of the article and conducted the bibliographic research. S. M. and H. K. contributed to an early draft, and G. G. M. wrote the manuscript. G. G. M. and S. M. made figures. All the authors participated in the revision of the manuscript.

## Conflicts of interest

There are no conflicts to declare.

## Supplementary Material
